# Regulatory interplay between nitric oxide and heme in redox signaling and inflammation

**DOI:** 10.1016/j.redox.2026.104126

**Published:** 2026-03-16

**Authors:** Pooja Pradhan, Roberta Foresti, Roberto Motterlini, Stephan Immenschuh

**Affiliations:** a Institute of Transfusion Medicine and Transplant Engineering, Hannover Medical School, Hannover, Germany; b University Paris-Est Créteil, INSERM, IMRB, Créteil, F-94010, France

**Keywords:** Heme, Hemoproteins, Inflammation, iNOS, Macrophages, Nitric oxide

## Abstract

Nitric oxide (NO) is a key signaling gas that is involved in a wide range of physiological and pathophysiological processes. NO signaling is closely linked to its interactions with heme, an abundant iron-containing tetrapyrrole in the organism. While heme plays vital roles as a prosthetic group in hemoproteins, it can be toxic in its ‘free’, non-protein-bound form. The chemical and structural characteristics of NO-heme binding in heme-nitrosyl complexes have been extensively characterized in earlier research. Recent studies have provided novel insights on how NO-heme interactions affect key functions of the cell and activities of subcellular organelles such as mitochondria. Notably, the NO-heme network plays a crucial immunomodulatory role in inflammatory responses of macrophages, a major cell population of the innate immune system. Upon immunological activation, these cells generate large amounts of NO through activation of the inducible nitric oxide synthase (iNOS), which contributes to killing of bacteria and modulating of inflammation. NO generates microbicidal pro-oxidant peroxynitrite, which in turn activates feedback loops that provide autoprotection to macrophages. Interestingly, the dynamic interaction between NO and heme adds to the complex control of various heme-containing enzymes involved in inflammation and cellular oxidative stress adaptation. NO interacts with heme both directly and indirectly through multiple biochemical reactions, such as S-nitrosylation of cysteine residues and allocation of heme into specific hemoproteins. The current review summarizes key aspects of the regulatory interplay between NO and heme, highlighting its functional consequences in health and disease. A particular emphasis is on the significance of the NO-heme network in inflammation, especially its role in macrophages.

## 1 Introduction

Nitric oxide (NO) is a signaling gas produced by three distinct isoforms of NO synthase (NOS) - the constitutively expressed endothelial (eNOS or NOS-3), neuronal (nNOS or NOS-1) NO synthases, and the inducible isoform (iNOS or NOS-2) [1]. In addition, NO can also be generated through a reverse pathway from nitrate (NO_3_^−^) and nitrite (NO_2_^−^) [2]. NO generated by eNOS primarily acts as an endothelium-derived signaling factor, with NO diffusing from blood vessels to the underlying smooth muscles to induce vasorelaxation. Similarly, NO formed by nNOS, serves as a neurotransmitter involved in synaptic signaling and in the regulation of neurovascular blood flow. Both eNOS and nNOS generate low or physiological NO concentrations that are in the pM to low nM range. In contrast, iNOS is up-regulated in macrophages by inflammatory mediators such as lipopolysaccharide (LPS) [3] and produces high or immunological levels of NO in the μM range [4,5]. High levels of NO facilitate the antimicrobial functions of macrophages [6,7] via formation of bactericidal compounds, which also require autoprotective defense mechanisms in these cells [8,9]. Moreover, iNOS-derived NO promotes immune responses through the release of cytokines [10].

Since the discovery of NO and the elucidation of its biological importance in the 1970s and 1980s [[11], [12], [13]] - an achievement that led to Robert Furchgott, Ferid Murad and Louis Ignarro to receive the Nobel Prize in Physiology or Medicine in 1998 - extensive research has explored the mechanisms underlying the actions of NO. These functions are primarily exerted through NO's high binding affinity to the heme prosthetic groups of several key proteins such as soluble guanylate cyclase (sGC), iNOS and mitochondrial complexes. Additionally, NO-mediated signaling also occurs through a post-translational modification known as S-nitrosylation [14], which targets cysteine residues in structural and functional proteins such as hemoglobin [15], glyceraldehyde-3-phosphate dehydrogenase (GAPDH) [16] and N-Methyl-d-Aspartate (NMDA) [17] receptor as well as small peptides like glutathione (GSH) [18,19]. In addition to NO production, the immune system also dynamically regulates heme, an iron-containing tetrapyrrole that serves as a prosthetic group of numerous hemoproteins involved in essential physiological functions, including oxygen storage and transport, electron transfer in the mitochondrial respiratory chain and drug metabolism [20,21]. Early seminal studies in endothelial cells established the dual nature of heme in pathophysiology, demonstrating its capacity to both exacerbate oxidant-mediated cellular damage and simultaneously trigger protective feedback loops, such as induction of heme oxygenase-1 (HO-1) [22,23]. The regulation of heme synthesis, degradation, and trafficking becomes especially significant in inflammatory conditions, because heme homeostasis and hemoprotein activity are closely linked to immune function. Moreover, extracellular free heme, which is released during hemolysis, can be toxic through the Fenton reaction, an iron (II)-catalyzed oxidation process activated in the presence of hydrogen peroxide that triggers pro-oxidant, pro-inflammatory and cytotoxic effects [24]. While free iron can catalyze the Fenton reaction to produce hydroxyl radicals [25], the interaction of hydrogen peroxide with intact heme leads to the formation of highly reactive ferryl or ferryl radical cation species [26,27]. This distinction is important, as each iron species dictates the specific oxidative pathways and downstream signaling effects during inflammatory stress. The detailed mechanisms governing the interplay between NO and heme in inflammatory responses is not yet fully understood. Thus, elucidating the mutual regulation of these two molecules is critical for unraveling the molecular processes that drive both inflammatory activation and resolution.

In the current review, we will discuss how NO and heme interact under physiological and inflammatory conditions, with an emphasis on the biochemical, cellular, and systemic implications of this relationship. We will focus on immunological relevant concentrations of NO and examine how NO and heme together regulate the balance between host defense and pathological inflammation, while also identifying key gaps in our current understanding of this intriguing research area.

## 2 Direct binding of NO with hemoproteins and heme: implications for signaling

NO binds directly to the iron atom in the heme moiety of hemoproteins through metal nitrosylation [28], modulating their redox state and catalytic functions. This interaction is central to a variety of physiological and pathological processes including immune regulation, vasodilation and cellular respiration [29]. The specificity and outcome of NO-heme binding are influenced by factors such as the redox state of heme iron, NO concentrations, ligand environment, and protein conformation. Understanding the structural and functional consequences of this binding is crucial for elucidating how NO modulates diverse cellular pathways. The following sections briefly discuss key aspects of NO-heme interactions, including their structural characteristics, and the role of NO in activating hemoproteins such as sGC.

### 2.1 Redox chemistry and structural dynamics of NO-heme binding

The reactivity of NO with heme is fundamentally governed by the redox state of the iron center, which typically exists in either ferrous (Fe^2+^), or ferric (Fe^3+^) form [30]. This redox-dependent interaction is both chemically selective and thermodynamically favorable allowing NO to bind effectively with heme when the iron is in its ferrous state [28,31,32]. Under these conditions, NO binding is largely reversible [30] and depends on the concentration of NO, with faster association rates observed in the presence of excess NO. However, binding of NO to ferric heme involves a lower affinity and a faster dissociation rate. Structurally, NO binds to ferrous heme in either a linear Fe(II)–N–O configuration, which is energetically favored, although a less stable Fe(II)–*O*–N geometry has also been observed [33,34].

Moreover, NO binding to heme can result in either a five-coordinate Fe(II)–NO or a six-coordinate His-Fe(II)–NO complex [28,34]. In the six-coordinate state, the distal histidine ligand remains bound but may be sterically or electronically destabilized, whereas in the five-coordinate form, NO replaces the distal ligand entirely, often inducing subtle conformational changes in the distal heme pocket [35,36]. These coordination states are also observed in sGC [37,38] and cytochrome *c* [39]. The conformational transitions induced by NO binding are fundamental to the regulation of heme protein activity under both physiological and stress-related conditions. Recent reviews on heme-binding proteins (HBPs) have provided extensive insights into the complex reactivity of NO with the heme iron center [40,41].

### 2.2 sGC activation

**Fig. 1:**
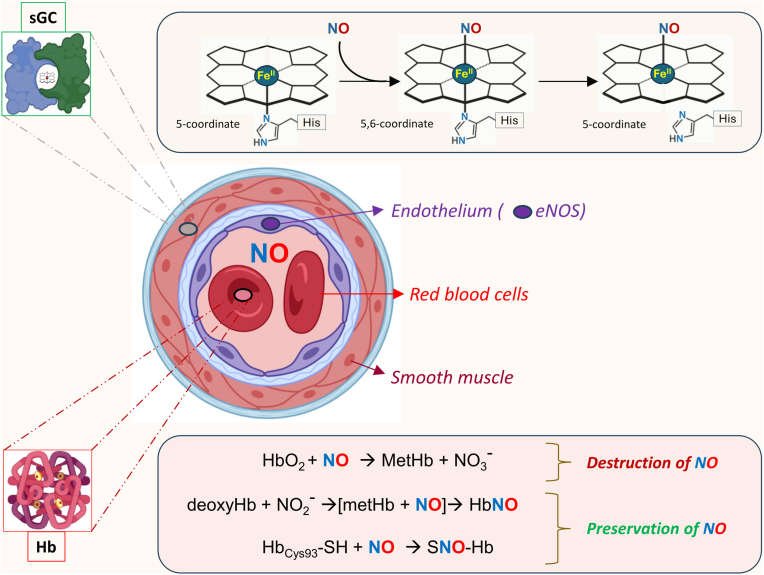
**Interaction of NO with key heme-dependent proteins. Top.** This scheme illustrates how activation of sGC by NO involves the initial NO binding to the heme moiety to form a six-coordinate complex (6C–NO) that transitions to a fully active state (5C–NO). **Bottom**. Differential NO binding to heme iron or β93 cysteine in hemoglobin determines the fate of NO that could be either destroyed to nitrate or preserved in the form of to S-Nitrosohemoglobin to facilitate vasodilation. See text for more details. sGC, soluble guanylate cyclase; Hb, hemoglobin; MetHb, methemoglobin; NO_3_-, nitrite; deoxyHb, deoxygenated hemoglobin; SNO-Hb, S-Nitrosohemoglobin.

One of the best-characterized examples of the NO interaction with heme is sGC [[42], [43], [44], [45], [46]], a key NO sensor. sGC is a cytosolic hemoprotein [[47], [48], [49]], that plays a central role in NO-dependent signaling including vasodilation and neurotransmission [50,51]. The molecular mechanism of NO-induced activation of sGC is complex and not yet fully understood. Evidence suggests that NO binding to sGC occurs in two sequential steps: (i) an initial rapid phase involving the formation of a six-coordinate His-Fe(II)–NO complex, followed by (ii) a slower phase characterized by the dissociation of the proximal His105-Fe(II) bond, resulting in the formation of a five-coordinate Fe(II)–NO species [37,[52], [53], [54]] (Fig. 1, top). Once formed, the Fe-NHis bond is broken and a conformational change activates the cyclase catalytic domain of sGC to catalyze the conversion of GTP to cyclic GMP (cGMP), a key second messenger involved in the regulation of vasodilation and cardiac contractility [55]. Both activation and deactivation of sGC are regulated by NO concentrations [[56], [57], [58]]. This mechanism is well established in endothelial and smooth muscle cells, and efforts in understanding the NO-heme/sGC-cGMP pathway have led to the development of sGC activators for treating diseases including hypertension, heart failure and chronic renal disease [[59], [60], [61], [62], [63]]. However, the specific regulatory roles of NO-hemoprotein interactions in macrophages are less clearly defined.

### 2.3 NO binding to hemoglobin and the NO-ferroheme complex

Because NO is continuously produced by the endothelium and accumulation of high NO concentrations cause toxicity, studies indicated that hemoproteins such as hemoglobin or myoglobin have a role in NO scavenging to limit its bioavailability [[64], [65], [66]]. In red blood cells (RBCs) and blood circulation, hemoglobin and unbound heme appear to be also important for NO transport. A series of studies have demonstrated that following NO inhalation or nitrite administration to human volunteers, a nitrosyl (heme)hemoglobin complex is the predominant species that transports and delivers NO to mediate vasodilatation [67,68]. A small fraction of NO can also bind cysteine 93 in the β-chain of hemoglobin to form an S-nitrosothiol (SNO) that may contribute to transport of NO in the circulation [67,69] (Fig. 1, bottom). However, βCys93 is susceptible to irreversible oxidation to cysteic acid, promoting ferryl heme and globin radical formation [70]. Moreover, methemoglobin formed through interactions with NO or nitrite is prone to releasing heme moieties [71].

Furthermore, while NO signaling was traditionally thought to be mediated via gaseous free NO, it seems that, instead of NO gas, a stable molecular NO-ferroheme complex may be directly released from NOS [72,73]. This hypothesis is supported by both earlier and recent studies showing that NO-ferroheme is a more efficient activator of sGC compared to free NO [43,74,75]. Furthermore, the stronger bond within the NO-ferroheme complex allows it to function as a stable, mobile signaling entity [28,76]. Interestingly, recent data from in vitro studies on primary human RBCs and mouse models show that labile ferric heme, not incorporated into a protein, but solubilized into RBC membranes or bound to serum albumin, can stabilize NO in a ferrous heme-nitrosyl complex via a nitrosylation reaction catalyzed by thiol groups [77]. The authors of this work proposed that this complex could function as a signaling molecule, where NO-ferroheme could be transferred from RBC membranes to albumin, then to apo-sGC leading to its activation. This cascade ultimately leads to inhibition of platelet activation and causes vasodilation in mice. Specifically, GSH promotes this process by reducing nitrosylation of ferric heme to facilitate the formation of ferroheme. The importance of this complex in physiological or pathophysiological conditions remains to be established, but these findings provide additional information on the signaling activity of NO that is dependent on binding to the heme molecule. A significant knowledge gap also persists regarding whether the NO-ferroheme complex offers enhanced resistance to NO-induced toxicity. Nevertheless, existing studies show that NO-ferroheme can be easily taken up by cells and that the presence of NO increases heme cellular accumulation compared to heme alone [[78], [79], [80], [81]]. Therefore, these findings suggest that there is a reciprocal regulation between NO and heme: on the one side, heme is a target of NO to execute its messenger functions, while on the other side, NO can potentially affect the regulatory role of heme in cells and tissues.

## 3 NO-dependent regulation of enzymatic heme synthesis and degradation

### 3.1 Heme biosynthesis: regulation of ALAS-1 and FECH by NO

**Fig. 2:**
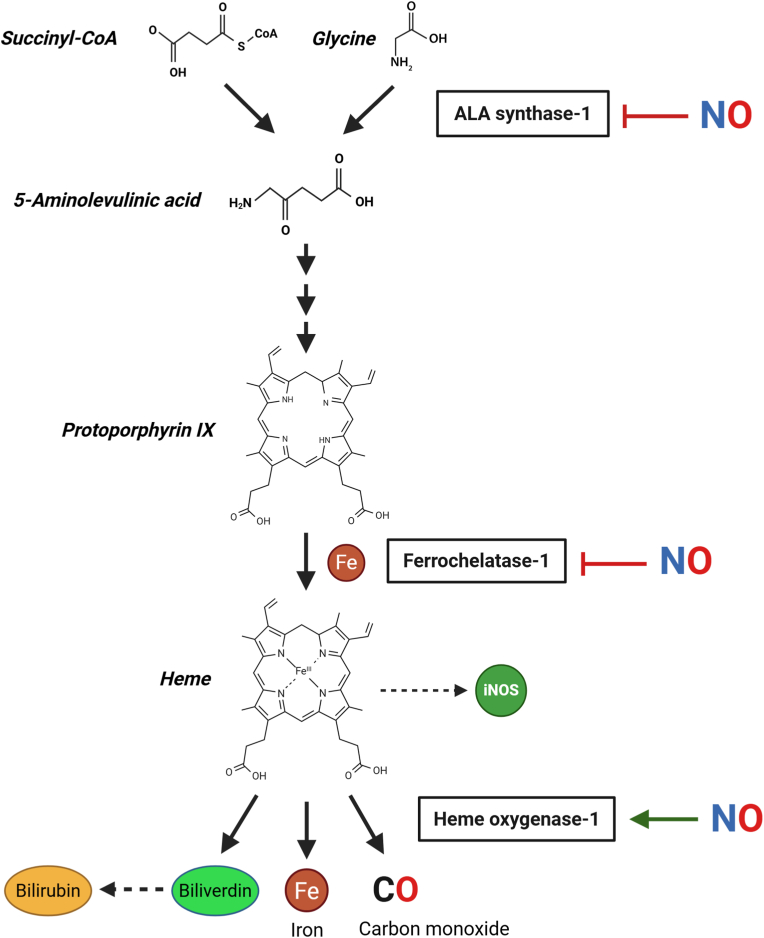
**How NO can regulate heme biosynthesis and degradation.** The schematic diagram illustrates which enzymes in the heme biosynthesis pathway are inhibited by NO and how NO can act as an inducer of HO-1, the rate-limiting step of heme degradation to produce CO and biliverdin, which is further converted to bilirubin. See text for more details.

Heme synthesis takes place in the mitochondria and in the cytosol via several metabolic steps [82] that can be influenced by NO. Succinyl -CoA from the tricarboxylic acid cycle and the amino acid glycine condense to form 5′-aminolevulinic acid (ALA), a rate-limiting reaction catalyzed by aminolevulinic acid synthase (ALAS). Two ALA molecules then condense in the cytosol to form porphobilinogen. This pyrrole ring unites to produce a four-pyrrole species that is transformed into various porphyrinogen intermediates and coproporphyrinogen, and finally reenters the mitochondria to form protoporphyrin IX. The last step of heme synthesis is catalyzed by ferrochetalase (FECH), which inserts ferrous iron into protoporphyrin IX [83,84] (Fig. 2). Heme is then distributed to different HBPs through a regulated trafficking network that has been the subject of extensive investigation [85].

Studies conducted in hepatocytes producing endogenous NO, either via stimulation with a cytokine mixture or exposure to a NO donor, revealed that the activities of ALAS and FECH are significantly inhibited in a NO-dependent manner. However, the mechanisms of inhibition differ between these two enzymes: ALAS activity is reduced due to NO-induced heme liberation, in line with the well-known feedback mechanism whereby free heme acts on ALAS to block heme synthesis [86]. In contrast, purified bovine FECH activity appears to be directly inhibited by NO, possibly via interaction with its iron-sulfur cluster [87]. Other authors reported a similar effect in mouse peritoneal macrophages, where stimulation with interferon (IFN)-γ plus LPS induced NO production caused by a decreased FECH activity. In the same study, purified recombinant human FECH exposed to NO donors showed alterations in its absorbance spectrum, including the loss of a characteristic peak at 460 nm typical of iron-sulfur clusters [88]. NO also influences iron metabolism [89], which may further affect heme synthesis. However, the precise mechanisms of these interactions are still largely unknown.

### 3.2 Heme degradation: NO-dependent modulation of HO-1 expression

NO also plays a critical role in heme degradation through its effects on HO-1, the inducible isoform of HO, that is responsible for catalyzing the first step in heme catabolism to biliverdin, carbon monoxide (CO) and free iron. Several studies have demonstrated that NO donors or NO derived from iNOS can up-regulate HO-1 expression at the transcriptional level, primarily through the activation of redox-sensitive transcription factors such as nuclear factor erythroid 2-related factor 2 (Nrf2) and activating protein-1 (AP-1) [90,91]. Importantly, other reports have also highlighted a close relationship between NO synthases and HO-1 proteins. For instance, it has been hypothesized that high NO levels produced by iNOS up-regulate HO-1 in order to limit the availability of heme for NOS activity [92]. Under pro-inflammatory conditions, the NO-mediated HO-1 induction would serve to restrain excessive NO production and restore cellular homeostasis. This concept is supported by the temporal patterns of these responses, in which iNOS is initially induced, followed by a delayed increase in HO-1 expression accompanied by reduced NO generation [[93], [94], [95]]. NO can also increase the expression of other stress proteins, such as heat-shock protein (HSP)70 [96,97], indicating that NO in concentrations above the basal levels acts as a signaling molecule in the modulation of the tissue stress response, as discussed in more detail in a later chapter. This induction represents a cytoprotective mechanism whereby heme-mediated oxidative stress is reduced not only by HO-1 enzymatic activity that degrades free heme levels, but also by HO-1-derived metabolites such as biliverdin/bilirubin and CO that possess antioxidant and signaling properties. Indeed, overexpression of HO-1 in endothelial cells after treatment with NO donors conferred an enhanced resistance to hydrogen peroxide-mediated cellular injury [98].

It is interesting to note that the concomitant presence of NO and heme markedly enhances the induction of HO-1 in endothelial cells [79] and this effect has been shown to be accompanied by the production of bilirubin, due to a strong enhancement of heme incorporation into cells [99]. Interestingly, the amplification of HO-1 induction was also observed in endothelial cells co-exposed to NO donors and hemoglobins, which are the most important extracellular source of heme under oxidative, inflammatory as well as hemolytic conditions, where NO levels are also altered [81]. Because heme may form a complex with NO, the action of hemin was also compared to that of a pure heme-nitrosyl adduct, demonstrating that this complex elicited a greater heme incorporation into cells than hemin alone. These data suggest that NO, possibly via the formation of a heme-NO complex, can directly modulate the uptake of heme in cells, leading to augmented HO-1 induction and heme catalysis. Therefore, an understanding of how NO regulates the interaction and delivery of heme to heme-containing proteins and to heme-degrading enzymes will provide clues on the function of these proteins in physiological and pathophysiological conditions.

Conversely, independent studies indicated that high levels of NO seem to affect HO enzymes in a detrimental manner. In rat PC12 cells and transfected HEK293 cells, it was shown that the activity of HO-2, which is constitutively present and contains specific heme-binding sites that are independent from its heme catalytic activity, was inhibited by exposure to NO donors, while HO-1 remained unaffected [100]. Peroxynitrite, an oxidant that increases during inflammation following the reaction of NO with superoxide, also diminished HO in rat spleen and brain, although no distinction was made between the effects on HO-1 and HO-2 [101]. Although the mechanisms accounting for these NO-mediated actions have not been fully explored, it is particularly intriguing that HO-2 contains three cysteine residues proposed to modulate its heme affinity, whereas HO-1 completely lacks cysteines [102]. This structural distinction strongly suggests that HO-2 could be readily inactivated through cysteine oxidation or S-nitrosylation under conditions of oxidative or nitrosative stress. In contrast, the absence of cysteines in HO-1 allows it to retain full enzymatic activity, enabling the protein to continue performing its cytoprotective functions even during high levels of reactive oxygen species (ROS) and NO. These characteristics highlight HO-1 as a truly stress-resistant, inducible, and essential cytoprotective system, likely critical for cellular survival under extreme oxidative and nitrosative conditions.

## 4 NO as a modulator of intracellular heme allocation and the labile heme pool

NO is a key modulator of intracellular heme levels because it regulates trafficking and allocation of heme into various apo-hemoproteins [85,103]. Notably, the effects of NO are dose-dependent and, at low concentrations, NO facilitates the incorporation of heme into specific apo-hemoproteins, such as iNOS and sGC, promoting their maturation and functional activity [45,75]. In contrast, high NO levels can reversibly inhibit heme insertion into distinct apo-hemoproteins [104]. This inhibition can temporarily increase labile heme levels and modulate activity of hemoproteins during nitrosative stress. Moreover, recent findings suggest that inactivation of specific hemoproteins may be mediated via S-nitrosylation-dependent modifications [103] rather than loss of heme suggesting a role for post-translational modifications [87]. Due to the central role of iNOS in inflammatory macrophages, the crosstalk between NO and heme for iNOS regulation will be discussed below in more detail.

Heme chaperones such as GAPDH [105,106], progesterone receptor membrane component 2 (PGRMC2) [107], and the general molecular chaperone HSP90 [[103], [104], [105]] play critical roles for intracellular trafficking and allocation of heme into apo-hemoproteins. Heme chaperones have distinct functions, for example, the ATP-dependent chaperone HSP90 stabilizes protein folding during heme incorporation into apo-hemoproteins [103]. In contrast, GAPDH primarily acts as an intracellular heme transporter and mediates the transfer of *de novo* synthesized mitochondrial heme and that of exogenous heme into apo-hemoproteins [106]. Interestingly, chaperone activity of GAPDH is regulated by NO, with low NO levels promoting heme transfer from GAPDH to the hemoprotein and high NO levels favoring its transfer back from the hemoprotein to GAPDH [108]. The mitochondrial heme transporter FLVCR1b can also supply heme to GAPDH and thus regulate heme homeostasis [109]. The precise mechanisms, by which GAPDH acquires and transfers heme, and whether additional proteins are involved, remain to be determined. An overview on functions of heme chaperones has recently been given by Stuehr and colleagues [103].

### 4.1 Regulation of the cellular labile heme pool

Allocation of heme into intracellular apo-hemoproteins relies on the rapid mobilization of bioavailable heme from the so-called labile heme pool. The labile heme pool is the cellular fraction of exchangeable or regulatory heme, which is loosely and transiently bound to proteins and functions as an intermediary to facilitate allocation of heme into apo-hemoproteins [110]. Such concept has previously been proposed for the regulation of ALAS1 enzyme activity in a cell culture model of chicken embryo hepatocytes [111]. Studies on labile heme have been hampered by a lack of feasible methods for distinguishing labile heme from total heme in the cell [112]. In fact, most fluorometric and enzyme-based methods lack the sensitivity to detect the low, physiologically active concentrations of labile heme and to measure the small changes of its levels. However, recently developed approaches that apply fluorescence methods with plasmid-based heme sensors [80,113,114] or chemical probes like H-FluNox [115] have enabled the determination of intracellular labile heme levels with high sensitivity and specificity.

Notably, NO may also modulate the labile heme pool of the cell via active heme mobilization. For example, treatment with pharmacological NO donors can cause a transient elevation of intracellular heme levels in yeast and HeLa cells [80,115], but the physiological consequences of this NO-dependent heme mobilization are incompletely understood. Various factors such as protein-protein interactions, cellular compartmentalization or dynamic fluctuations of redox homeostasis may affect labile heme availability and distribution in the cell. Thus, it is an open question whether and how labile heme levels are modulated by heme buffering via heme chaperones or other HBPs, enzymatic degradation and shuttling into the extracellular environment. Finally, it has also been proposed that labile heme may serve as a source for forming an NO-ferroheme complex [72]. In conclusion, further studies are required to investigate the interplay between NO and labile heme in physiological and clinical relevant conditions.

## 5 The interplay between NO and heme in inflammatory macrophages

In the following, we will discuss how NO, heme and the mutual interplay of the two compounds can modulate macrophage functions. Major tasks of macrophages are killing of phagocytosed microorganisms and orchestrating of inflammatory responses. Due to the major antimicrobial and immunomodulatory effects of NO, iNOS is a key player of these immunological events [6,116] that is induced by prototypical pro-inflammatory stimuli such as IFN-γ, LPS or tumor necrosis factor-α (TNF-α) [8]. Moreover, it modulates the activities of inflammatory enzymes such as cyclooxygenase-2 [117] and NADPH oxidase [[118], [119], [120]].

### 5.1 Feedback regulation of iNOS activity by NO and heme

The enzymatic activity of iNOS is governed via NO- and heme-dependent feedback loops in macrophages [121]. Similar to other NO synthases, iNOS requires heme as a prosthetic group of its oxygenase domain [122,123] for catalyzing the conversion of l-arginine into NO [124]. In contrast to eNOS and nNOS, iNOS binds calmodulin permanently to ensure high and continuous NO production [125]. iNOS enzyme activity depends on the reduction of heme iron via NADPH-derived electrons [126]. Notably, NO can inhibit heme insertion into iNOS via a feedback mechanism to prevent excessive NO production [104,127]. Therefore, during prolonged exposure to high levels of NO, iNOS activity is repressed and the electron flow shifts towards formation of superoxide (discussed in more detail below). The functional role of heme is well known for the regulation of iNOS enzyme activity [127,128], but not for that of iNOS gene expression. Elucidation of the latter mechanisms may not only give insights into the modulation of inflammatory responses, but may also help to develop novel therapeutic approaches via targeting of iNOS.

In combination with superoxide, NO forms peroxynitrite, a highly reactive nitrogen species (RNS) [129]. Peroxynitrite causes nitration and oxidation of lipids, proteins and nucleic acids, which in turn lead to cytoxicity [130]. Moreover, excessive peroxynitrite formation amplifies inflammatory signaling and tissue injury [131]. Consistent with these effects, in vivo studies in various murine disease models have highlighted the detrimental role of iNOS in inflammatory macrophages [132]. Interestingly, peroxynitrite also increases HO-1 expression, suggesting that this inducible heme degrading enzyme is sensitive to different NO-derived species [133].

In inflammation, high levels of NO also lead to S-nitrosylation, a reversible post-translational modification, in which NO is covalently attached to the thiol side chain of specific cysteine residues [134]. This modification orchestrates diverse immunomodulatory responses in macrophages [135]. For example, S-nitrosylation modulates NF-κB, a central transcriptional activator of pro-inflammatory genes. Specifically, NO modifies critical cysteine residues in the p50 and p65 subunits of NF-κB [136,137], and also stabilizes IκB, the inhibitor of NF-κB via preventing its degradation [138]. Thus, NO is also involved in a negative feedback loop for up-regulation of its own enzymatic synthesis, but also of other NF-κB-dependent genes [139,140]. In addition, S-nitrosylation inhibits caspase-1 to alleviate inflammasome activation and maturation of IL-1β and IL-18 [141] and also regulates hypoxia-inducible factor-1 (HIF-1α), a key nuclear factor for controlling the expression of oxygen-dependent genes [142,143]. Finally, modification of actin-regulatory proteins alters cytoskeletal dynamics that affect phagocytotic activity and antigen-processing during inflammatory activation of macrophages [144]. Together, S-nitrosylation appears to be a key feedback mechanism in inflammatory macrophages.

### 5.2 Extracellular heme and activation of TLR4 in macrophages

Extracellular heme has signaling functions that are distinct to those of intracellular heme. In particular, free, i.e. non-protein bound heme is a damage-associated molecular pattern (DAMP) that is critical for the pathophysiological events, which are caused by hemolysis and/or ischemia-reperfusion injury [145]. Extracellular heme exerts its effects primarily through binding to and activation of Toll-like receptor 4 (TLR4) in macrophages [146]. However, the role of heme in TLR4 signaling is not fully understood because findings are conflicting depending on distinct experimental models. For example, studies in kidney injury models using the TLR4 inhibitor TAK-242 or TLR4 knockout mice revealed contradictory results [147]. Moreover, heme and LPS appear to induce markedly different gene expression profiles in human and mouse macrophages [148] suggesting distinct inflammatory responses in the two species (discussed in more detail below). It is also noteworthy that the pro-inflammatory effects of heme depend on its interactions with HBPs, because binding to various HBPs mediate different cellular responses [149,150]. This experimental variability highlights a paradox in heme biology as demonstrated by Vallelian and Schaer [149]. Notably, clinical observations do not support the ‘heme-*as*-DAMP’ model because hemolysis or sterile tissue injuries with significant heme release do not necessarily cause a systemic inflammatory response in humans. An emerging concept, however, is that extracellular heme can be a functional ‘second hit’ in experimental settings of inflammatory stimulation. Extracellular heme can synergize with pro-inflammatory stimuli such as LPS to amplify inflammatory responses [151] and/or NOD-, LRR- and pyrin domain-containing protein (NLRP)3 inflammasome activation [152]. The second hit hypothesis may also extend to cardiovascular disorders, in which free heme can exacerbate pre-existing pro-inflammatory endothelial activation of blood vessels. Such mechanisms may occur in disorders such as sickle cell disease and atypical hemolytic uremic syndrome [153].

## 6 Autoprotection mechanisms in macrophages against the toxicity of NO and heme

The killing of microorganisms after phagocytosis by macrophages is associated with the generation of large amounts of ROS and RNS that require efficient antioxidant defense mechanisms. The Keap1/Nrf2 system plays a key role in autoprotection via induction of defense genes that restore cellular homeostasis [[154], [155], [156]]. In normal cellular conditions, Nrf2 is continuously produced and sequestered via its repressor protein Keap1 that facilitates Nrf2 ubiquitination and degradation [157]. However, in pro-oxidant conditions, modifications of Keap1 cysteine residues impair its E3 ligase activity and derepress Nrf2 that translocates into the nucleus. In the nucleus, Nrf2 binds to antioxidant response elements (AREs), and mediates the induction of numerous inducible antioxidant and detoxifying genes such as HO-1, NAD(P)H:quinone oxidoreductase 1 (NQO1), and glutathione S-transferases [158]. In mouse bone marrow-derived macrophages (BMDMs), Nrf2 has been shown to be stabilized via activation of Mst1/2 kinases, which are mammalian homologues of Drosophila Hippo [156]. Mst1/2 have been shown to be activated via TLR4 signaling leading to the generation of large amounts of ROS during an antimicrobial response [159].

Self-protection in activated macrophages is also provided by the GSH system that controls the intracellular redox balance to maintain homeostasis. GSH functions as a ROS scavenger and may also counteract S-nitrosylation [160]. The latter is also achieved via the activity of denitrosylases, which inactivate SNO modifications in proteins that mediate the restoration of normal cellular function [161]. For example, the thioredoxin reductase system can denitrosylate and reconstitute the function of NF-κB, thereby restoring inflammatory signaling [162].

**Fig. 3:**
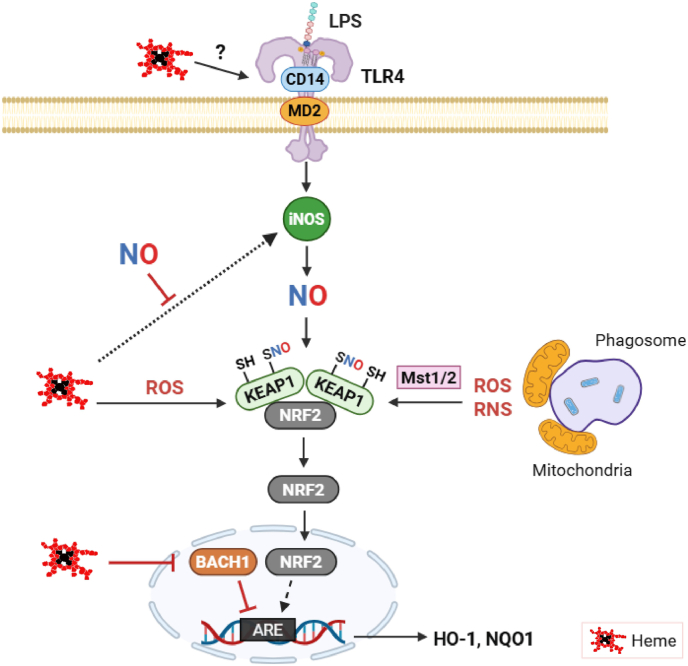
**The Keap1/Nrf2 system: autoprotection in macrophages against NO and heme toxicity.** Oxidative and nitrosative stress generated induced by inflammation (LPS/TLR4) triggers protective pathways that restore cellular homeostasis. NO promotes activation of the Keap1/Nrf2 pathway through S-nitrosylation of Keap1, allowing Nrf2 to induce antioxidant and cytoprotective genes such as HO-1, NQO1, and ferritin. Heme can also stimulate this pathway indirectly through its pro-oxidant properties and through regulation of the nuclear repressor BACH1. Elevated intracellular levels of heme promote BACH1 degradation, enabling induction of HO-1, which degrades excess heme and limits toxicity. Together, these mechanisms coordinate redox control, iron sequestration, and detoxification to protect inflammatory macrophages. See text for more details.

NO can also activate the Keap1/Nrf2 pathway through S-nitrosylation of Keap1 cysteine residues, that in turn, leads to the production of protective antioxidant and repair proteins [163] (Fig. 3). Beyond its direct signaling roles, NO influences iron sequestration by transcriptionally regulating ferritin expression, largely through activation of the Nrf2 pathway [164]. As activated macrophages are a key source of secreted ferritin during the acute-phase response [165], this NO-dependent regulation serves to limit iron availability for the Fenton reaction and restrict pathogen access to iron [166]. Interestingly, heme also affects the Keap1/Nrf2 system via its pro-oxidant properties that can lead to an antioxidant defense response. Moreover, heme also modulates gene expression via the heme sensor protein and nuclear repressor BTB-and–CNC–homology 1 (BACH1), which is a functional repressor of antioxidant genes by binding to Maf recognition elements under homeostatic conditions [167]. When intracellular heme levels increase, BACH1 is degraded and enables Nrf2 binding to AREs, which then activates gene expression of HO-1 [168]. Ultimately, induction of HO-1 leads to enzymatic degradation of excess heme, which serves as a negative feedback loop to restore cellular homeostasis. Heme-dependent regulation of BACH1 also appears to be important in inflammation, because BACH1-deficient mice exhibit increased resistance to both infectious and sterile inflammatory challenges [[169], [170], [171]]. Additionally, macrophages from mice with genetic BACH1-deficiency display reduced iNOS induction upon inflammatory stimulation [172]. Whether this attenuation of iNOS expression is caused by decreased intracellular heme levels in BACH1−/− macrophages is not known. It also remains to be clarified whether NO can directly modulate BACH1 activity and whether NO-ferroheme may activate the Nrf2/BACH1 axis.

At this stage, it is important to point out that the interpretation of findings regarding the effects of NO on activation of the cellular stress response should be approached with caution. Most experiments performed in vitro are conducted at atmospheric O_2_ (20%), which does not reflect the much lower “physiological” oxygen concentrations found in tissues in vivo (typically <10%) [173,174]. Thus, the artifactual hyperoxic environment commonly used in cell culture experiments can significantly influence NO bioavailability, its competition with O_2_ for heme-binding sites, and the resulting cellular signaling. For instance, RAW264.7 macrophages cultured at 20% O_2_ have been reported to exhibit a pro-oxidant phenotype, characterized by lower levels of reduced glutathione, increased production of ROS, and higher expression of Nrf2, HO-1, and NQO1 compared with macrophages continuously maintained at 5% O_2_. Interestingly, following treatment with LPS, the increase in several pro-inflammatory markers, including iNOS expression, nitrite production and TNF-α, was much lower in macrophages cultured at 5% O_2_ [175]. These observations, together with other studies [176,177], emphasize the importance of oxygen availability in cellular adaptation to oxidative stress and inflammatory stimuli, which may help explain some of the divergent results observed between cell-based models and animal studies.

## 7 NO, heme and mitochondria

**Fig. 4:**
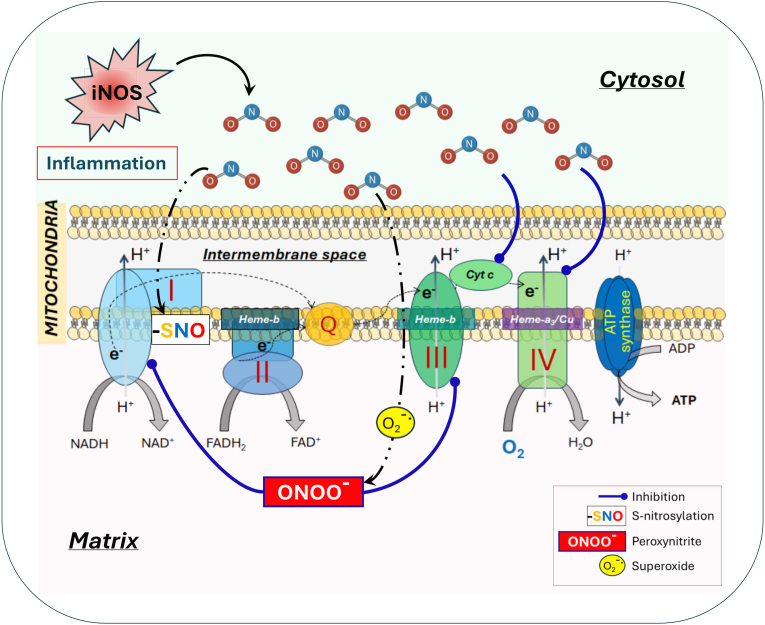
**Regulation of mitochondrial metabolism by NO in macrophages.** NOmodulates mitochondrial function through interactions with respiratory chain complexes, including reversible inhibition of cytochrome *c* oxidase (complex IV) and modulation of complexes I and III. These effects suppress OXPHOS, promote glycolysis, alter TCA cycle metabolite levels, and influence ROS production. The outcome is context-dependent, affecting macrophage metabolic state, inflammatory phenotype, and repolarization capacity. Post-translational modifications such as S-nitrosylation contribute to NO-mediated regulation of metabolic enzymes. See text for more details.

NO has emerged as a critical regulator of mitochondrial metabolism, largely through its ability to interact with respiratory chain complexes (Fig. 4). One of the most well-known effects of NO on the respiratory chain is the reversible inhibition of cytochrome *c* oxidase (complex IV), a hemoprotein located in the inner mitochondrial membrane [178]. By binding to the heme-copper center of the enzyme, NO competitively displaces molecular oxygen and blocks the terminal step of the electron transport chain [[179], [180], [181], [182], [183], [184]]. Consequently, mitochondrial respiration is suppressed, leading to a metabolic shift from oxidative phosphorylation (OXPHOS) towards aerobic glycolysis, a hallmark of pro-inflammatory macrophage activation [185]. This is accompanied by reduced ATP production and elevated ROS generation. The outcome of this interaction depends on the local oxygen concentrations, activity of iNOS and also the redox state of complex IV [186]. This metabolic shift may be further regulated by other signaling molecules. For example, in mouse BMDMs IL-10 secreted by LPS-stimulated cells has been shown to employ a metabolic control loop that regulates NO production [187]. The authors demonstrate that LPS-induced glycolytic flux controls IL-10 production, which causes the shift towards glycolysis by preventing the suppression of OXPHOS by NO. The interaction of NO with mitochondria may also be a determinant of the inflammatory status in macrophages. Van den Bossche et al. reported that BMDMs stimulated with LPS + IFN-γ to acquire a pro-inflammatory phenotype, exhibit mitochondrial dysfunction that was partially dependent on NO overproduction [188]. Interestingly, pro-inflammatory macrophages were unable to switch to an anti-inflammatory profile upon incubation with IL-4, a classical cytokine used to induce alternative activated macrophages. However, inhibiting iNOS activity with the compound 1400W significantly restored mitochondrial OXPHOS and concomitantly improved the repolarization capacity of pro-inflammatory cells. In contrast, NO counteracted the activation of the NLRP3 inflammasome and iNOS deficiency in mouse BMDMs resulted in mitochondrial damage and increase mitochondrial ROS production in macrophages primed with LPS and subsequently treated with ATP [189]. Even if the two articles cited above did not look at the same inflammatory parameters in macrophages and adopted completely different protocols of macrophage stimulation, together they highlight that the interaction of NO and mitochondria is context-dependent and can lead to opposing effects on markers of inflammation.

In addition, NO influences tricarboxylic acid cycle metabolites such as citrate, succinate, and itaconate [190,191], and modulates NADPH dehydrogenase (complex I) and ubiquinol-cytochrome c reductase (complex III) [192] through mechanisms involving peroxynitrite formation [193], S-nitrosylation and tyrosine nitration. S-nitrosylation modulates immunometabolism by modifying metabolic enzymes such as components of the pyruvate dehydrogenase complex, thereby altering metabolic flux and shaping macrophage inflammatory states [194] (Fig. 4).

NO also regulates mitochondrial biogenesis and dynamics, primarily through the induction of peroxisome proliferator-activated receptor gamma coactivator 1-alpha (PGC-1α) [[195], [196], [197]], with additional modulation of the redox-sensitive pathways such as HO-1/CO axis [198]. Through these mechanisms, NO not only supports energy supply but also facilitates cellular adaptation to oxidative and inflammatory stress. While physiological levels of NO promote mitochondrial turnover and cellular adaptation to stress, prolonged and excessive NO exposure results in mitochondrial hyperpolarization [199], and structural damage. Such dysregulation exacerbates inflammatory responses and increases susceptibility to cellular injury, in part by interfering with signaling pathways such as AMPK, sirtuin, and NF-κB, which are central to mitochondrial quality control and cellular stress responses.

Emerging evidence indicates that exogenous/free heme significantly impacts mitochondrial and cellular metabolism, with direct consequences on immune and tissue health. A recent study has demonstrated that heme exposure impairs oxidative phosphorylation and shifts cellular metabolism toward glycolysis in human retinal pigment epithelial cells [200]. This process is driven by the nuclear translocation of HIF-1α and the subsequent up-regulation of hypoxia-driven genes such as VEGFA and GLUT1, suggesting that heme triggers a pseudo-hypoxic response through mitochondrial interference. In mouse macrophages, heme exposure has been shown to reduce mitochondrial mass and dynamics [201], while simultaneously diminishing oxygen consumption and ATP levels. These metabolic shifts correlate with increased inflammation and limited efferocytosis. Notably, rescuing mitochondrial biogenesis and dynamics by increasing PGC1α and PPARγ expression improved ATP levels, reduced inflammation and recovered efferocytosis. Similarly, mitochondrial damage was observed in Kupffer cells in an animal model of sepsis induced by cecal ligation and puncture, where the elevated heme levels correlated with loss of macrophages and senescence [202]. The contribution of heme in this effect was demonstrated by experiments showing that hemopexin, a heme scavenger, effectively prevented Kupffer cell death and senescence, accompanied by enhanced bacterial clearance.

Heme has also been shown to trigger early mitochondria ROS generation, which is strongly implicated in cytokine production by macrophages [203]. Therefore, heme in non-physiological levels interacts and modulates mitochondria, most likely in an unfavorable manner. Whether NO and heme reciprocally influence each other in the modulation of mitochondria metabolism and inflammation is a new area of investigation and some initial clues that this may occur under inflammatory stimulation are emerging [148].

## 8 NO, heme and cell death

Initiation and progression of inflammation are intimately linked with various pathways of regulated cell death (RCD) [204]. Different forms of RCD (for a review see Ref. [205]) can determine the natural course of an inflammatory response [206,207]. Apoptosis, the first described form of RCD, has initially been considered as a non-inflammatory form of programmed cell death [208], but has recently also been demonstrated to mediate inflammation in certain conditions [209] and to also exhibit functional links with non-apoptotic forms of RCD [210]. In contrast to apoptosis, non-apoptotic forms of RCD, such as necroptosis (a regulated form of necrosis) and ferroptosis can initiate inflammation through the release of DAMPs [211] that activate pattern recognition receptors (PRRs) [212]. It should be noted that while necrosis is often used to describe passive, unregulated cell death, pathways like necroptosis and ferroptosis represent genetically encoded, regulated processes. In this section, we will discuss how NO and heme can modulate three selected forms of RCD - apoptosis, ferroptosis and PANoptosis, a recently characterized type of programmed cell death that integrates features of pyroptosis, apoptosis, and necroptosis [213] (Table 1), and how this may regulate the ensuing inflammatory response.

**Table 1:** Dual actions and context-dependent functions of NO and heme in regulating cell death pathways.

Effect	Mechanism
**NO**	

**Pro-apoptotic**	- **High NO concentrations:** - Activation of BAX and cytochrome *c* release [214] - Degradation of the anti-apoptotic protein MCL-1 via ASK1-JNK1 signaling axis [215] - Accumulation of tumor suppressor protein p53 ([216]) - S-nitrosylation of NF-κB [217]

**Anti-apoptotic**	**Low NO concentrations:** - Direct S-nitrosylation and inhibition of caspase −3, −8, and −9 (216, 219) - Activation of pro-survival signaling pathways such as the ERK [218] and AKT pathways [219]

**Anti-ferroptotic**	**Low to moderate NO concentrations:** - Abort lipid peroxidation chain reaction [220] - Competition with O_2_ for access to the catalytic site of 15-lipoxygenase [221] - Direct interaction with and termination of lipid peroxyl radicals [222]

**Heme**	

**Pro-apoptotic/Pro-necroptotic**	**Excess free heme:** - Catalyzes the production of ROS via the Fenton reaction, leading to oxidative stress and damage to lipid, proteins, and DNA [24] - Induces programmed necrosis through TLR4/MyD88-dependent TNF production and RIP1/RIP3 protein signaling [223]

**Pro-ferroptotic**	**Heme degradation via HO-1:** - The released pro-oxidant Fe^2+^ can accumulate beyond the buffering capacity of ferritin, leading to ROS overload and lipid peroxidation [[224], [225], [226]]

**Pro-PANoptotic**	**In combination with PAMPs/TNF:** - Activates NLRP12 –PANoptosome [202,213] - The PANoptosome is a multi-protein complex that drives a coordinated cell death response integrating pyroptosis (GSDMD/E cleavage), apoptosis (caspase-3/7 activation), and necroptosis (RIPK3/MLKL phosphorylation) [227]

NO is a key modulator of apoptosis in macrophages with concentration-dependent effects [228,229]. On the one hand, low physiological levels of NO levels provide anti-apoptotic effects and promote cell proliferation via activation of sGC, cGMP-dependent pathways and inhibition of caspase activation [230]. These anti-apoptotic NO effects are largely mediated by S-nitrosylation of caspases, which suppresses their proteolytic activity to block apoptosis [[231], [232], [233]]. On the other hand, high concentrations of NO can promote apoptosis by disrupting mitochondrial metabolism [199,234,235] and inducing endoplasmic reticulum (ER) stress, a mechanism demonstrated in RAW267.4 macrophages [236]. In contrast to NO, the role of heme in apoptosis is less well-characterized, as high concentrations of free heme exert pro-oxidant effects that lead to non-apoptotic cell death, such as necrosis [223] and ferroptosis [224]. However, as heme is also a potent inducer of ER stress [[237], [238], [239]], a potential interplay may exist, in which the convergence of heme and NO exacerbates cellular damage by co-triggering ER stress-mediated apoptotic pathways.

Accumulating experimental evidence suggests that a fine-tuned balance between NO and heme affects the cellular redox status and iron metabolism, thereby determining susceptibility to oxidative damage and ferroptosis. An important player of this balance is HO-1, the enzymatic activity of which can increase intracellular levels of iron, a key mediator of ferroptosis required for the activity of lipoxygenase-dependent lipid peroxidation [[224], [225], [226]]. When HO-1-dependent heme catabolism is deregulated, excess iron may amplify lipid peroxidation, and increase cellular susceptibility to ferroptosis. Moreover, BACH1 may enhance ferroptosis by reducing the expression of iron regulatory and antioxidant genes [240]. Notably, NO has been shown to provide protection against ferroptosis [220,241]. In fact, it can directly inhibit lipid peroxidation chain reactions through its scavenger function [220], and also via S-nitrosylation of proteins that counteract ferroptosis. For example, this protective function has been demonstrated in host defense, where NO released by macrophages diffuses to adjacent epithelial cells and prevents ferroptosis [242].

In PANoptosis, heme has been shown to exacerbate LPS or bacterial-induced activation of this pathway through the activation of NLRP12 [202,213]. The study investigated different combinations of pattern-associated molecular patterns (PAMPs) and DAMPs for their ability to cause cell death in macrophages. Only the unique combination of heme with TLR ligands (LPS and Pam3CSK4 to mimic bacterial infection, and polyinosinic:polycytidylic acid and resiquimod to mimic viral infection) induced robust cell death. In contrast, no cell death was observed when TLR ligands were co-incubated with other DAMPs, such as HMGB1. Whether NLRP12 is a direct sensor of heme, and how the balance between its sequestration and release regulates NLRP12 activation is not known. Moreover, the potential role of NO for NLRP12 modulation in macrophages requires further studies.

In conclusion, the interactions of NO and heme with cell death are complex and context-dependent, with significant variations across different cell types and microenvironments. For instance, while NO typically decreases mitochondrial membrane potential (ΔΨm) to induce apoptosis in numerous cell types, such as thymocytes [235] and natural killer cells [243], the opposite was observed in activated RAW264.7 macrophages [199]. Similar cell type-specific variations are evident during ferroptosis, because classically activated macrophages exhibit enhanced resistance to this form of cell death due to robust NO production, which effectively inhibits lipid peroxidation [241]. This resistance may represent a crucial self-protective adaptation, allowing macrophages to maintain function and clear pathogens without succumbing to NO-induced toxicity.

## 9 Species-specificity of the inflammatory response in mouse and human macrophages

Mice play a key role as experimental tools in biomedicine. In particular, observations from preclinical studies with mouse models are important before translation into the clinic. While studies in mice have led to major basic and clinically relevant discoveries in the field of immunology, a number of inter species-specific differences between mouse and human have been reported [244]. An important example for distinct immune-regulatory mechanisms between mouse and human has been shown in a comparative study on various inflammatory disorders. In fact, by directly comparing the genomic responses in mouse models that closely mimic human inflammatory conditions such as trauma, burns and endotoxemia, the authors of this work revealed only poor correlations [245]. A comparative study on TLR4-dependent inflammatory responses of primary murine and human macrophages also highlighted a complex regulatory pattern with distinct phenotypes, which may partly be explained by differences in the promoter regions of inducible pro-inflammatory genes [246]. Similarly, LPS-dependent inflammatory activation can cause different bioenergetics adaptive patterns in primary mouse and human macrophages. While LPS stimulation of mouse macrophages leads to a significant shift towards glycolysis and a marked impairment of OXPHOS, also known as metabolic reprogramming, LPS-activated human macrophages primarily rely on OXPHOS for energy production and do not undergo a metabolic shift toward glycolysis [247]. In the following, we will discuss how NO and heme can modulate inflammation via distinct effects in mouse and human macrophages.

**Fig. 5:**
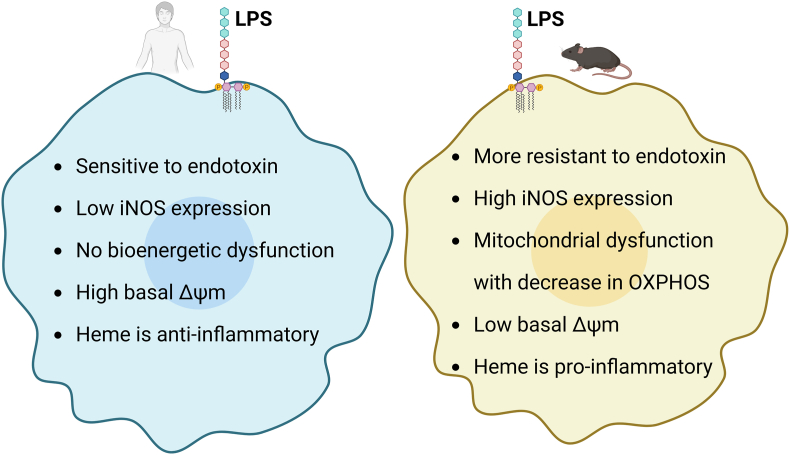
**Species-specific differences in inflammatory response and metabolic profiles of human and mouse macrophages.** Mouse and human macrophages exhibit distinct inflammatory and metabolic profiles. LPS stimulation shifts mouse macrophages toward glycolysis with high iNOS/NO expression, whereas human macrophages primarily rely on OXPHOS and show low iNOS levels. Differences in NO availability, ΔΨm, and regulatory pathways, including heme signaling, contribute to species-specific responses. These disparities suggest to take caution when extrapolating findings from mouse models of inflammation to human conditions. See text for more details.

In contrast to inflammatory mouse macrophages, in which iNOS is highly expressed, human macrophages only display low expression levels of this enzyme, which may partly be due to epigenetic repression of the iNOS gene locus [248,249]. However, the issue of iNOS expression and NO production in human macrophages remains a subject of ongoing debates [250,251], and a recent study on species-specific differences between mouse and human for arginase-1 expression has demonstrated an additional level of complexity [252]. Intriguingly, NO has been shown to be critical for mitochondrial functions in mouse and human macrophages [253]. Consistent with this work, we have recently shown that availability of NO can also influence the regulatory effects of heme on inflammatory phenotypes of mouse and human macrophages [148]. Specifically, treatment of human cells with an NO donor exacerbated LPS-induced pro-inflammatory cytokines, causing heme to lose its anti-inflammatory properties and acquire a pro-inflammatory phenotype. An explanation for this species-specific regulatory variation may be the differential levels of the ΔΨm in mouse and human macrophages [247]. As discussed above, high concentrations of NO are closely associated with ΔΨm depolarization, which further links NO availability to species-specific mitochondrial and inflammatory responses. Thus, different levels of NO production may affect antibacterial immunological reactions, modulation of inflammatory responses and affect the course of diseases. For example, mice exhibit significantly higher resistance to endotoxin compared to humans [244]. Further distinctions also extend to gene expression signatures and surface protein profiles, with many commonly used markers for macrophage polarization displaying divergent expression patterns in human and mouse cells [254]. Finally, comparative analysis of TLR pathways in human and mouse macrophages revealed species-specific preferences in reliance on distinct IL-1-receptor-associated kinase (IRAK) proteins [255]. Fig. 5 summarizes the major differences in inflammatory and metabolic profiles between human and mouse macrophages.

In summary, the distinct regulatory role of NO and heme in inflammatory mouse and human macrophages may be explained by differential evolutionary developments of the innate immune system in rodents and primates. These disparities underscore the need for caution when translating findings from murine macrophage studies to inflammatory responses in human diseases.

## 10 Conclusion and outlook

**Fig. 6:**
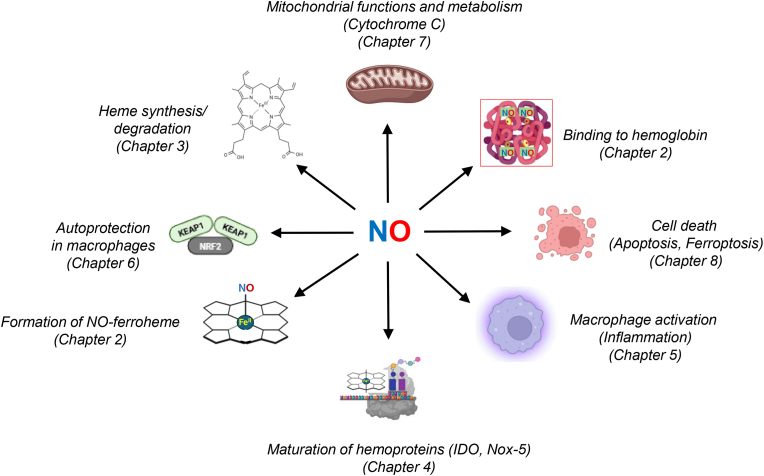
**Differential interactions of NO with heme in the cell.** This scheme illustrates a summary of the main intracellular targets and pathways that can be affected or altered by NO within the context of macrophages inflammatory and metabolic responses.

The interplay between NO and heme is essential for maintaining cellular homeostasis, particularly in inflammatory conditions. Heme plays a central role in NO signaling by reversibly binding NO and catalyzing reactions that promote its scavenging or synthesis. Thus, heme creates a feedback system that prevents pathological levels of NO production. Macrophages are key regulators in this redox-dependent interplay due to their high capacity for NO production. The balance between NO- and heme-mediated signaling in these cells determines a spectrum of outcomes, ranging from immune homeostasis and pathogen clearance to inflammation, mitochondrial dysfunction, and cell death (Fig. 6). To counteract the inherent toxicity of both NO and heme, macrophages have evolved sophisticated mechanisms of autoprotection. In addition to its signaling functions, NO also acts as a respiratory modulator by competing with oxygen for mitochondrial heme centers, leading to inhibition of mitochondrial respiration. The consequences of this inhibition are dependent on the concentration, duration of exposure and cell type, all of which ultimately determine whether cells survive or die. The implications of NO-heme interactions in various disease models, where heme release is a common denominator (e.g., hemolysis in sickle cell disease, infection and sepsis, and organ ischemia-reperfusion) warrant further investigation. While, the NO-heme interactions discussed here primarily focus on macrophages, similar redox crosstalk is likely relevant in other cell types, such as vascular endothelial cells, which also participate in inflammatory signaling. Because vascular and immune cells frequently interact at sites of tissue injury, NO-heme mediated metabolic shifts in one cell type may profoundly influence the inflammatory phenotype of the surrounding microenvironment. Given the significant species-specific differences and the ongoing debate regarding iNOS expression in humans, studies employing human-relevant models are urgently needed to better predict clinical outcomes. Future investigations should focus on the NO-ferroheme complex as a key signaling entity and examine how fluctuations in the labile heme pool regulate its formation and effects on immune cell activation. Beyond the canonical signaling functions of NO-heme interactions (e.g. heme release and NO-mediated enzyme inhibition or activation during oxidative stress), future studies should also address how NO and heme coordinately regulate inflammatory responses and how inflammatory mediators reciprocally influence this critical NO-heme axis.

## CRediT authorship contribution statement

**Pooja Pradhan:** Conceptualization, Writing – original draft. **Roberta Foresti:** Conceptualization, Writing – original draft, Writing – review & editing. **Roberto Motterlini:** Conceptualization, Writing – original draft, Writing – review & editing. **Stephan Immenschuh:** Conceptualization, Writing – original draft, Writing – review & editing.

## Declaration of competing interest

The authors declare that they have no known competing financial interests or personal relationships that could have appeared to influence the work reported in this paper.

## Data Availability

No data was used for the research described in the article.

## References

[1] Forstermann U., Sessa W.C. (2012). Nitric oxide synthases: regulation and function. Eur. Heart J..

[2] Carlstrom M., Weitzberg E., Lundberg J.O. (2024). Nitric oxide signaling and regulation in the cardiovascular system: recent advances. Pharmacol. Rev..

[3] Stuehr D.J., Marletta M.A. (1985). Mammalian nitrate biosynthesis: mouse macrophages produce nitrite and nitrate in response to Escherichia coli lipopolysaccharide. Proc. Natl. Acad. Sci. U. S. A..

[4] Thomas D.D., Ridnour L.A., Isenberg J.S., Flores-Santana W., Switzer C.H., Donzelli S., Hussain P., Vecoli C., Paolocci N., Ambs S., Colton C.A., Harris C.C., Roberts D.D., Wink D.A. (2008). The chemical biology of nitric oxide: implications in cellular signaling. Free Radic. Biol. Med..

[5] Hall C.N., Garthwaite J. (2009). What is the real physiological NO concentration in vivo?. Nitric Oxide.

[6] Hibbs J.B., Taintor R.R., Vavrin Z., Rachlin E.M. (1988). Nitric oxide: a cytotoxic activated macrophage effector molecule. Biochem. Biophys. Res. Commun..

[7] Stuehr D.J., Nathan C.F. (1989). Nitric oxide. A macrophage product responsible for cytostasis and respiratory inhibition in tumor target cells. J. Exp. Med..

[8] MacMicking J., Xie Q.W., Nathan C. (1997). Nitric oxide and macrophage function. Annu. Rev. Immunol..

[9] Watanabe S., Alexander M., Misharin A.V., Budinger G.R.S. (2019). The role of macrophages in the resolution of inflammation. J. Clin. Investig..

[10] Cinelli M.A., Do H.T., Miley G.P., Silverman R.B. (2020). Inducible nitric oxide synthase: regulation, structure, and inhibition. Med. Res. Rev..

[11] Arnold W.P., Mittal C.K., Katsuki S., Murad F. (1977). Nitric oxide activates guanylate cyclase and increases guanosine 3':5'-cyclic monophosphate levels in various tissue preparations. Proc. Natl. Acad. Sci. U. S. A..

[12] Ignarro L.J., Byrns R.E., Buga G.M., Wood K.S. (1987). Endothelium-derived relaxing factor from pulmonary artery and vein possesses pharmacologic and chemical properties identical to those of nitric oxide radical. Circ. Res..

[13] Furchgott R.F., Zawadzki J.V. (1980). The obligatory role of endothelial cells in the relaxation of arterial smooth muscle by acetylcholine. Nature.

[14] Martinez-Ruiz A., Lamas S. (2004). S-nitrosylation: a potential new paradigm in signal transduction. Cardiovasc. Res..

[15] Jia L., Bonaventura C., Bonaventura J., Stamler J.S. (1996). S-nitrosohaemoglobin: a dynamic activity of blood involved in vascular control. Nature.

[16] Mohr S., Stamler J.S., Brune B. (1996). Posttranslational modification of glyceraldehyde-3-phosphate dehydrogenase by S-nitrosylation and subsequent NADH attachment. J. Biol. Chem..

[17] Choi Y.B., Tenneti L., Le D.A., Ortiz J., Bai G., Chen H.S., Lipton S.A. (2000). Molecular basis of NMDA receptor-coupled ion channel modulation by S-nitrosylation. Nat. Neurosci..

[18] Stamler J.S., Jaraki O., Osborne J., Simon D.I., Keaney J., Vita J., Singel D., Valeri C.R., Loscalzo J. (1992). Nitric oxide circulates in mammalian plasma primarily as an S-nitroso adduct of serum albumin. Proc. Natl. Acad. Sci. U. S. A..

[19] Clancy R.M., Levartovsky D., Leszczynska-Piziak J., Yegudin J., Abramson S.B. (1994). Nitric oxide reacts with intracellular glutathione and activates the hexose monophosphate shunt in human neutrophils: evidence for S-nitrosoglutathione as a bioactive intermediary. Proc. Natl. Acad. Sci. U. S. A..

[20] Ponka P. (1999). Cell biology of heme. Am. J. Med. Sci..

[21] Voltarelli V.A., Alves de Souza R.W., Miyauchi K., Hauser C.J., Otterbein L.E. (2023). Heme: the lord of the iron ring. Antioxidants.

[22] Balla G., Vercellotti G.M., Muller-Eberhard U., Eaton J., Jacob H.S. (1991). Exposure of endothelial cells to free heme potentiates damage mediated by granulocytes and toxic oxygen species. Lab. Invest..

[23] Balla J., Jacob H.S., Balla G., Nath K., Eaton J.W., Vercellotti G.M. (1993). Endothelial-cell heme uptake from heme proteins: induction of sensitization and desensitization to oxidant damage. Proc. Natl. Acad. Sci. U. S. A..

[24] Kumar S., Bandyopadhyay U. (2005). Free heme toxicity and its detoxification systems in human. Toxicol. Lett..

[25] Wardman P., Candeias L.P. (1996). Fenton chemistry: an introduction. Radiat. Res..

[26] Harel S., Kanner J. (1988). The generation of ferryl or hydroxyl radicals during interaction of haemproteins with hydrogen peroxide. Free Radic. Res. Commun..

[27] Patel R.P., Svistunenko D.A., Darley-Usmar V.M., Symons M.C., Wilson M.T. (1996). Redox cycling of human methaemoglobin by H2O2 yields persistent ferryl iron and protein based radicals. Free Radic. Res..

[28] Hunt A.P., Lehnert N. (2015). Heme-nitrosyls: electronic structure implications for function in biology. Acc. Chem. Res..

[29] Ignarro L.J. (2002). Nitric oxide as a unique signaling molecule in the vascular system: a historical overview. J. Physiol. Pharmacol..

[30] Cooper C.E. (1999). Nitric oxide and iron proteins. Biochim. Biophys. Acta.

[31] Tawa M., Okamura T. (2022). Factors influencing the soluble guanylate cyclase heme redox state in blood vessels. Vasc. Pharmacol..

[32] Goodrich L.E., Paulat F., Praneeth V.K., Lehnert N. (2010). Electronic structure of heme-nitrosyls and its significance for nitric oxide reactivity, sensing, transport, and toxicity in biological systems. Inorg. Chem..

[33] Nutt D.R., Karplus M., Meuwly M. (2005). Potential energy surface and molecular dynamics of MbNO: existence of an unsuspected FeON minimum. J. Phys. Chem. B.

[34] De Simone G., di Masi A., Sbardella D., Ascenzi P., Coletta M. (2024). Nitric oxide binding geometry in Heme-proteins: relevance for signal transduction. Antioxidants.

[35] Lawson D.M., Stevenson C.E., Andrew C.R., Eady R.R. (2000). Unprecedented proximal binding of nitric oxide to heme: implications for guanylate cyclase. EMBO J..

[36] Praneeth V.K., Neese F., Lehnert N. (2005). Spin density distribution in five- and six-coordinate iron(II)-porphyrin NO complexes evidenced by magnetic circular dichroism spectroscopy. Inorg. Chem..

[37] Zhao Y., Brandish P.E., Ballou D.P., Marletta M.A. (1999). A molecular basis for nitric oxide sensing by soluble guanylate cyclase. Proc. Natl. Acad. Sci. U. S. A..

[38] Martin E., Berka V., Sharina I., Tsai A.L. (2012). Mechanism of binding of NO to soluble guanylyl cyclase: implication for the second NO binding to the heme proximal site. Biochemistry.

[39] Andrew C.R., George S.J., Lawson D.M., Eady R.R. (2002). Six- to five-coordinate heme-nitrosyl conversion in cytochrome c' and its relevance to guanylate cyclase. Biochemistry.

[40] Lehnert N., Kim E., Dong H.T., Harland J.B., Hunt A.P., Manickas E.C., Oakley K.M., Pham J., Reed G.C., Alfaro V.S. (2021). The biologically relevant coordination chemistry of iron and nitric oxide: electronic structure and reactivity. Chem. Rev..

[41] Foley E.L., Hvitved A.N., Eich R.F., Olson J.S. (2022). Mechanisms of nitric oxide reactions with globins using mammalian myoglobin as a model system. J. Inorg. Biochem..

[42] Ohlstein E.H., Wood K.S., Ignarro L.J. (1982). Purification and properties of heme-deficient hepatic soluble guanylate cyclase: effects of heme and other factors on enzyme activation by NO, NO-heme, and protoporphyrin IX. Arch. Biochem. Biophys..

[43] Ignarro L.J., Adams J.B., Horwitz P.M., Wood K.S. (1986). Activation of soluble guanylate cyclase by NO-hemoproteins involves NO-heme exchange. Comparison of heme-containing and heme-deficient enzyme forms. J. Biol. Chem..

[44] Underbakke E.S., Iavarone A.T., Marletta M.A. (2013). Higher-order interactions bridge the nitric oxide receptor and catalytic domains of soluble guanylate cyclase. Proc. Natl. Acad. Sci. U. S. A..

[45] Ghosh A., Stasch J.P., Papapetropoulos A., Stuehr D.J. (2014). Nitric oxide and heat shock protein 90 activate soluble guanylate cyclase by driving rapid change in its subunit interactions and heme content. J. Biol. Chem..

[46] Schrammel A., Behrends S., Schmidt K., Koesling D., Mayer B. (1996). Characterization of 1H-[1,2,4]oxadiazolo[4,3-a]quinoxalin-1-one as a heme-site inhibitor of nitric oxide-sensitive guanylyl cyclase. Mol. Pharmacol..

[47] Gerzer R., Radany E.W., Garbers D.L. (1982). The separation of the heme and apoheme forms of soluble guanylate cyclase. Biochem. Biophys. Res. Commun..

[48] Karow D.S., Pan D., Davis J.H., Behrends S., Mathies R.A., Marletta M.A. (2005). Characterization of functional heme domains from soluble guanylate cyclase. Biochemistry.

[49] Ignarro L.J., Wood K.S., Wolin M.S. (1982). Activation of purified soluble guanylate cyclase by protoporphyrin IX. Proc. Natl. Acad. Sci. U. S. A..

[50] Yang X.M., Philipp S., Downey J.M., Cohen M.V. (2005). Postconditioning's protection is not dependent on circulating blood factors or cells but involves adenosine receptors and requires PI3-kinase and guanylyl cyclase activation. Basic Res. Cardiol..

[51] Penna C., Cappello S., Mancardi D., Raimondo S., Rastaldo R., Gattullo D., Losano G., Pagliaro P. (2006). Post-conditioning reduces infarct size in the isolated rat heart: role of coronary flow and pressure and the nitric oxide/cGMP pathway. Basic Res. Cardiol..

[52] Pyriochou A., Papapetropoulos A. (2005). Soluble guanylyl cyclase: more secrets revealed. Cell. Signal..

[53] Cary S.P., Winger J.A., Derbyshire E.R., Marletta M.A. (2006). Nitric oxide signaling: no longer simply on or off. Trends Biochem. Sci..

[54] Russwurm M., Koesling D. (2004). NO activation of guanylyl cyclase. EMBO J..

[55] Denninger J.W., Marletta M.A. (1999). Guanylate cyclase and the .NO/cGMP signaling pathway. Biochim. Biophys. Acta.

[56] Horst B.G., Marletta M.A. (2018). Physiological activation and deactivation of soluble guanylate cyclase. Nitric Oxide.

[57] Sayed N., Baskaran P., Ma X., van den Akker F., Beuve A. (2007). Desensitization of soluble guanylyl cyclase, the NO receptor, by S-nitrosylation. Proc. Natl. Acad. Sci. U. S. A..

[58] Schulman I.H., Hare J.M. (2012). Regulation of cardiovascular cellular processes by S-nitrosylation. Biochim. Biophys. Acta.

[59] Behrends S. (2003). Drugs that activate specific nitric oxide sensitive guanylyl cyclase isoforms independent of nitric oxide release. Curr. Med. Chem..

[60] Evgenov O.V., Pacher P., Schmidt P.M., Hasko G., Schmidt H.H., Stasch J.P. (2006). NO-independent stimulators and activators of soluble guanylate cyclase: discovery and therapeutic potential. Nat. Rev. Drug Discov..

[61] Stasch J.P., Pacher P., Evgenov O.V. (2011). Soluble guanylate cyclase as an emerging therapeutic target in cardiopulmonary disease. Circulation.

[62] Benza R.L., Grunig E., Sandner P., Stasch J.P., Simonneau G. (2024). The nitric oxide-soluble guanylate cyclase-cGMP pathway in pulmonary hypertension: from PDE5 to soluble guanylate cyclase. Eur. Respir. Rev..

[63] Stasch J.P., Schmidt P.M., Nedvetsky P.I., Nedvetskaya T.Y., H S.A., Meurer S., Deile M., Taye A., Knorr A., Lapp H., Muller H., Turgay Y., Rothkegel C., Tersteegen A., Kemp-Harper B., Muller-Esterl W., Schmidt H.H. (2006). Targeting the heme-oxidized nitric oxide receptor for selective vasodilatation of diseased blood vessels. J. Clin. Investig..

[64] Doyle M.P., Hoekstra J.W. (1981). Oxidation of nitrogen oxides by bound dioxygen in hemoproteins. J. Inorg. Biochem..

[65] Gow A.J., Luchsinger B.P., Pawloski J.R., Singel D.J., Stamler J.S. (1999). The oxyhemoglobin reaction of nitric oxide. Proc. Natl. Acad. Sci. U. S. A..

[66] Flogel U., Merx M.W., Godecke A., Decking U.K., Schrader J. (2001). Myoglobin: a scavenger of bioactive NO. Proc. Natl. Acad. Sci. U. S. A..

[67] Gladwin M.T., Ognibene F.P., Pannell L.K., Nichols J.S., Pease-Fye M.E., Shelhamer J.H., Schechter A.N. (2000). Relative role of heme nitrosylation and beta-cysteine 93 nitrosation in the transport and metabolism of nitric oxide by hemoglobin in the human circulation. Proc. Natl. Acad. Sci. U. S. A..

[68] Cosby K., Partovi K.S., Crawford J.H., Patel R.P., Reiter C.D., Martyr S., Yang B.K., Waclawiw M.A., Zalos G., Xu X., Huang K.T., Shields H., Kim-Shapiro D.B., Schechter A.N., Cannon R.O., Gladwin M.T. (2003). Nitrite reduction to nitric oxide by deoxyhemoglobin vasodilates the human circulation. Nat. Med..

[69] Stamler J.S., Simon D.I., Osborne J.A., Mullins M.E., Jaraki O., Michel T., Singel D.J., Loscalzo J. (1992). S-nitrosylation of proteins with nitric oxide: synthesis and characterization of biologically active compounds. Proc. Natl. Acad. Sci. U. S. A..

[70] Alayash A.I. (2021). betaCysteine 93 in human hemoglobin: a gateway to oxidative stability in health and disease. Lab. Invest..

[71] Bunn H.F., Jandl J.H. (1966). Exchange of heme among hemoglobin molecules. Proc. Natl. Acad. Sci. U. S. A..

[72] Kleschyov A.L. (2017). The NO-heme signaling hypothesis. Free Radic. Biol. Med..

[73] Lundberg J.O., Weitzberg E. (2022). Nitric oxide signaling in health and disease. Cell.

[74] Craven P.A., DeRubertis F.R. (1978). Restoration of the responsiveness of purified guanylate cyclase to nitrosoguanidine, nitric oxide, and related activators by heme and hemeproteins. Evidence for involvement of the paramagnetic nitrosyl-heme complex in enzyme activation. J. Biol. Chem..

[75] Dai Y., Faul E.M., Ghosh A., Stuehr D.J. (2022). NO rapidly mobilizes cellular heme to trigger assembly of its own receptor. Proc. Natl. Acad. Sci. U. S. A..

[76] Kleschyov A.L., Zhuge Z., Schiffer T.A., Guimaraes D.D., Zhang G., Montenegro M.F., Tesse A., Weitzberg E., Carlstrom M., Lundberg J.O. (2023). NO-ferroheme is a signaling entity in the vasculature. Nat. Chem. Biol..

[77] DeMartino A.W., Poudel L., Dent M.R., Chen X., Xu Q., Gladwin B.S., Tejero J., Basu S., Alipour E., Jiang Y., Rose J.J., Gladwin M.T., Kim-Shapiro D.B. (2023). Thiol-catalyzed formation of NO-ferroheme regulates intravascular NO signaling. Nat. Chem. Biol..

[78] Sweeny E.A., Hunt A.P., Batka A.E., Schlanger S., Lehnert N., Stuehr D.J. (2021). Nitric oxide and heme-NO stimulate superoxide production by NADPH oxidase 5. Free Radic. Biol. Med..

[79] Foresti R., Hoque M., Bains S., Green C.J., Motterlini R. (2003). Haem and nitric oxide: synergism in the modulation of the endothelial haem oxygenase-1 pathway. Biochem. J..

[80] Hanna D.A., Harvey R.M., Martinez-Guzman O., Yuan X., Chandrasekharan B., Raju G., Outten F.W., Hamza I., Reddi A.R. (2016). Heme dynamics and trafficking factors revealed by genetically encoded fluorescent heme sensors. Proc. Natl. Acad. Sci. U. S. A..

[81] Foresti R., Bains S., Sulc F., Farmer P.J., Green C.J., Motterlini R. (2006). The interaction of nitric oxide with distinct hemoglobins differentially amplifies endothelial heme uptake and heme oxygenase-1 expression. J. Pharmacol. Exp. Therapeut..

[82] Ajioka R.S., Phillips J.D., Kushner J.P. (2006). Biosynthesis of heme in mammals. Biochim. Biophys. Acta.

[83] Ashenbrucker H., Cartwright G.E., Goldberg A., Wintrobe M.M. (1956). Studies on the biosynthesis of heme in vitro by avian erythrocytes. Blood.

[84] Dailey H.A., Dailey T.A., Wu C.K., Medlock A.E., Wang K.F., Rose J.P., Wang B.C. (2000). Ferrochelatase at the millennium: structures, mechanisms and [2Fe-2S] clusters. Cell. Mol. Life Sci..

[85] Donegan R.K., Moore C.M., Hanna D.A., Reddi A.R. (2019). Handling heme: the mechanisms underlying the movement of heme within and between cells. Free Radic. Biol. Med..

[86] Ponka P. (1997). Tissue-specific regulation of iron metabolism and heme synthesis: distinct control mechanisms in erythroid cells. Blood.

[87] Kim Y.M., Bergonia H.A., Muller C., Pitt B.R., Watkins W.D., Lancaster J.R. (1995). Loss and degradation of enzyme-bound heme induced by cellular nitric oxide synthesis. J. Biol. Chem..

[88] Furukawa T., Kohno H., Tokunaga R., Taketani S. (1995). Nitric oxide-mediated inactivation of mammalian ferrochelatase in vivo and in vitro: possible involvement of the iron-sulphur cluster of the enzyme. Biochem. J..

[89] Hentze M.W., Kuhn L.C. (1996). Molecular control of vertebrate iron metabolism: mrna-based regulatory circuits operated by iron, nitric oxide, and oxidative stress. Proc. Natl. Acad. Sci. U. S. A..

[90] Naughton P., Hoque M., Green C.J., Foresti R., Motterlini R. (2002). Interaction of heme with nitroxyl or nitric oxide amplifies heme oxygenase-1 induction: involvement of the transcription factor Nrf2. Cell. Mol. Biol..

[91] Immenschuh S., Tan M., Ramadori G. (1999). Nitric oxide mediates the lipopolysaccharide dependent upregulation of the heme oxygenase-1 gene expression in cultured rat Kupffer cells. J. Hepatol..

[92] Grion N., Repetto E.M., Pomeraniec Y., Calejman C.M., Astort F., Sanchez R., Pignataro O.P., Arias P., Cymeryng C.B. (2007). Induction of nitric oxide synthase and heme oxygenase activities by endotoxin in the rat adrenal cortex: involvement of both signaling systems in the modulation of ACTH-dependent steroid production. J. Endocrinol..

[93] Motterlini R., Foresti R., Bassi R., Calabrese V., Clark J.E., Green C.J. (2000). Endothelial heme oxygenase-1 induction by hypoxia. Modulation by inducible nitric-oxide synthase and S-nitrosothiols. J. Biol. Chem..

[94] Turcanu V., Dhouib M., Poindron P. (1998). Nitric oxide synthase inhibition by haem oxygenase decreases macrophage nitric-oxide-dependent cytotoxicity: a negative feedback mechanism for the regulation of nitric oxide production. Res. Immunol..

[95] Turcanu V., Dhouib M., Poindron P. (1998). Heme oxygenase inhibits nitric oxide synthase by degrading heme: a negative feedback regulation mechanism for nitric oxide production. Transplant. Proc..

[96] Xu Q., Hu Y., Kleindienst R., Wick G. (1997). Nitric oxide induces heat-shock protein 70 expression in vascular smooth muscle cells via activation of heat shock factor 1. J. Clin. Investig..

[97] Malyshev I., Malugin A.V., Golubeva L., Zenina T.A., Manukhina E.B., Mikoyan V.D., Vanin A.F. (1996). Nitric oxide donor induces HSP70 accumulation in the heart and in cultured cells. FEBS Lett..

[98] Motterlini R., Foresti R., Intaglietta M., Winslow R.M. (1996). NO-mediated activation of heme oxygenase: endogenous cytoprotection against oxidative stress to endothelium. Am. J. Physiol..

[99] Yee E.L., Pitt B.R., Billiar T.R., Kim Y.M. (1996). Effect of nitric oxide on heme metabolism in pulmonary artery endothelial cells. Am. J. Physiol..

[100] Ding Y., McCoubrey W.K., Maines M.D. (1999). Interaction of heme oxygenase-2 with nitric oxide donors. Is the oxygenase an intracellular 'sink' for NO?. Eur. J. Biochem..

[101] Kinobe R., Ji Y., Nakatsu K. (2004). Peroxynitrite-mediated inactivation of heme oxygenases. BMC Pharmacol..

[102] Varfaj F., Lampe J.N., Ortiz de Montellano P.R. (2012). Role of cysteine residues in heme binding to human heme oxygenase-2 elucidated by two-dimensional NMR spectroscopy. J. Biol. Chem..

[103] Stuehr D.J., Biswas P., Dai Y., Jayaram D.T., Sinha P.D., Sweeny E.A., Ghosh A. (2025). Key roles of GAPDH, Hsp90, and NO in heme trafficking. J. Inorg. Biochem..

[104] Waheed S.M., Ghosh A., Chakravarti R., Biswas A., Haque M.M., Panda K., Stuehr D.J. (2010). Nitric oxide blocks cellular heme insertion into a broad range of heme proteins. Free Radic. Biol. Med..

[105] Chakravarti R., Aulak K.S., Fox P.L., Stuehr D.J. (2010). GAPDH regulates cellular heme insertion into inducible nitric oxide synthase. Proc. Natl. Acad. Sci. U. S. A..

[106] Sweeny E.A., Singh A.B., Chakravarti R., Martinez-Guzman O., Saini A., Haque M.M., Garee G., Dans P.D., Hannibal L., Reddi A.R., Stuehr D.J. (2018). Glyceraldehyde-3-phosphate dehydrogenase is a chaperone that allocates labile heme in cells. J. Biol. Chem..

[107] Galmozzi A., Kok B.P., Kim A.S., Montenegro-Burke J.R., Lee J.Y., Spreafico R., Mosure S., Albert V., Cintron-Colon R., Godio C., Webb W.R., Conti B., Solt L.A., Kojetin D., Parker C.G., Peluso J.J., Pru J.K., Siuzdak G., Cravatt B.F., Saez E. (2019). PGRMC2 is an intracellular haem chaperone critical for adipocyte function. Nature.

[108] Biswas P., Palazzo J., Schlanger S., Jayaram D.T., Islam S., Page R.C., Stuehr D.J. (2024). Visualizing mitochondrial heme flow through GAPDH in living cells and its regulation by NO. Redox Biol..

[109] Jayaram D.T., Sivaram P., Biswas P., Dai Y., Sweeny E.A., Stuehr D.J. (2025). Heme allocation in eukaryotic cells relies on mitochondrial heme export through FLVCR1b to cytosolic GAPDH. Nat. Commun..

[110] Gallio A.E., Fung S.S., Cammack-Najera A., Hudson A.J., Raven E.L. (2021). Understanding the logistics for the distribution of heme in cells. JACS Au.

[111] Granick S., Sinclair P., Sassa S., Grieninger G. (1975). Effects by heme, insulin, and serum albumin on heme and protein synthesis in chick embryo liver cells cultured in a chemically defined medium, and a spectrofluorometric assay for porphyrin composition. J. Biol. Chem..

[112] Hopp M.T., Schmalohr B.F., Kuhl T., Detzel M.S., Wissbrock A., Imhof D. (2020). Heme determination and quantification methods and their suitability for practical applications and everyday use. Anal. Chem..

[113] Song Y., Yang M., Wegner S.V., Zhao J., Zhu R., Wu Y., He C., Chen P.R. (2015). A genetically encoded FRET sensor for intracellular heme. ACS Chem. Biol..

[114] Gallio A.E., Marson N.A., Heesom K.J., Lewis P.A., Alibhai D., Dugdale C.A., Herman A., Basran J., Hudson A.J., Raven E.L. (2025). An extended network for regulation of heme homeostasis in cells. Proc. Natl. Acad. Sci. U. S. A..

[115] Kawai K., Hirayama T., Imai H., Murakami T., Inden M., Hozumi I., Nagasawa H. (2022). Molecular imaging of labile Heme in living cells using a small molecule fluorescent probe. J. Am. Chem. Soc..

[116] Nathan C.F., Hibbs J.B. (1991). Role of nitric oxide synthesis in macrophage antimicrobial activity. Curr. Opin. Immunol..

[117] Salvemini D., Misko T.P., Masferrer J.L., Seibert K., Currie M.G., Needleman P. (1993). Nitric oxide activates cyclooxygenase enzymes. Proc. Natl. Acad. Sci. U. S. A..

[118] Jiang F., Roberts S.J., Datla Sr., Dusting G.J. (2006). NO modulates NADPH oxidase function via heme oxygenase-1 in human endothelial cells. Hypertension.

[119] Selemidis S., Dusting G.J., Peshavariya H., Kemp-Harper B.K., Drummond G.R. (2007). Nitric oxide suppresses NADPH oxidase-dependent superoxide production by S-nitrosylation in human endothelial cells. Cardiovasc. Res..

[120] Qian J., Chen F., Kovalenkov Y., Pandey D., Moseley M.A., Foster M.W., Black S.M., Venema R.C., Stepp D.W., Fulton D.J. (2012). Nitric oxide reduces NADPH oxidase 5 (Nox5) activity by reversible S-nitrosylation. Free Radic. Biol. Med..

[121] Chen Y., Panda K., Stuehr D.J. (2002). Control of nitric oxide synthase dimer assembly by a heme-NO-dependent mechanism. Biochemistry.

[122] Bredt D.S., Hwang P.M., Glatt C.E., Lowenstein C., Reed R.R., Snyder S.H. (1991). Cloned and expressed nitric oxide synthase structurally resembles cytochrome P-450 reductase. Nature.

[123] Siddhanta U., Wu C., Abu-Soud H.M., Zhang J., Ghosh D.K., Stuehr D.J. (1996). Heme iron reduction and catalysis by a nitric oxide synthase heterodimer containing one reductase and two oxygenase domains. J. Biol. Chem..

[124] Palmer R.M., Rees D.D., Ashton D.S., Moncada S. (1988). L-arginine is the physiological precursor for the formation of nitric oxide in endothelium-dependent relaxation. Biochem. Biophys. Res. Commun..

[125] Cho H.J., Xie Q.W., Calaycay J., Mumford R.A., Swiderek K.M., Lee T.D., Nathan C. (1992). Calmodulin is a subunit of nitric oxide synthase from macrophages. J. Exp. Med..

[126] Smith B.C., Underbakke E.S., Kulp D.W., Schief W.R., Marletta M.A. (2013). Nitric oxide synthase domain interfaces regulate electron transfer and calmodulin activation. Proc. Natl. Acad. Sci. U. S. A..

[127] Albakri Q.A., Stuehr D.J. (1996). Intracellular assembly of inducible NO synthase is limited by nitric oxide-mediated changes in heme insertion and availability. J. Biol. Chem..

[128] Stuehr D.J. (1997). Structure-function aspects in the nitric oxide synthases. Annu. Rev. Pharmacol. Toxicol..

[129] Koppenol W.H., Moreno J.J., Pryor W.A., Ischiropoulos H., Beckman J.S. (1992). Peroxynitrite, a cloaked oxidant formed by nitric oxide and superoxide. Chem. Res. Toxicol..

[130] Radi R. (2013). Peroxynitrite, a stealthy biological oxidant. J. Biol. Chem..

[131] Pacher P., Beckman J.S., Liaudet L. (2007). Nitric oxide and peroxynitrite in health and disease. Physiol. Rev..

[132] Farahani A., Farahani A., Kashfi K., Ghasemi A. (2025). Inducible nitric oxide synthase (iNOS): more than an inducible enzyme? Rethinking the classification of NOS isoforms. Pharmacol. Res..

[133] Foresti R., Sarathchandra P., Clark J.E., Green C.J., Motterlini R. (1999). Peroxynitrite induces haem oxygenase-1 in vascular endothelial cells: a link to apoptosis. Biochem. J..

[134] Heinrich T.A., da Silva R.S., Miranda K.M., Switzer C.H., Wink D.A., Fukuto J.M. (2013). Biological nitric oxide signalling: chemistry and terminology. Br. J. Pharmacol..

[135] Tran N., Mills E.L. (2024). Redox regulation of macrophages. Redox Biol..

[136] Marshall H.E., Stamler J.S. (2001). Inhibition of NF-kappa B by S-nitrosylation. Biochemistry.

[137] Kelleher Z.T., Matsumoto A., Stamler J.S., Marshall H.E. (2007). NOS2 regulation of NF-kappaB by S-nitrosylation of p65. J. Biol. Chem..

[138] Katsuyama K., Shichiri M., Marumo F., Hirata Y. (1998). NO inhibits cytokine-induced iNOS expression and NF-kappaB activation by interfering with phosphorylation and degradation of IkappaB-alpha. Arterioscler. Thromb. Vasc. Biol..

[139] Xie Q.W., Kashiwabara Y., Nathan C. (1994). Role of transcription factor NF-kappa B/Rel in induction of nitric oxide synthase. J. Biol. Chem..

[140] Taylor B.S., de Vera M.E., Ganster R.W., Wang Q., Shapiro R.A., Morris S.M., Billiar T.R., Geller D.A. (1998). Multiple NF-kappaB enhancer elements regulate cytokine induction of the human inducible nitric oxide synthase gene. J. Biol. Chem..

[141] Kim Y.M., Talanian R.V., Li J., Billiar T.R. (1998). Nitric oxide prevents IL-1beta and IFN-gamma-inducing factor (IL-18) release from macrophages by inhibiting caspase-1 (IL-1beta-converting enzyme). J. Immunol..

[142] Sandau K.B., Fandrey J., Brune B. (2001). Accumulation of HIF-1alpha under the influence of nitric oxide. Blood.

[143] Brune B., Zhou J. (2007). Hypoxia-inducible factor-1alpha under the control of nitric oxide. Methods Enzymol..

[144] Hagiwara M., Tada H., Matsushita K. (2025). Nitric oxide regulates phagocytosis through S-nitrosylation of Rab5. J. Biol. Chem..

[145] Soares M.P., Bozza M.T. (2016). Red alert: Labile heme is an alarmin. Curr. Opin. Immunol..

[146] Figueiredo R.T., Fernandez P.L., Mourao-Sa D.S., Porto B.N., Dutra F.F., Alves L.S., Oliveira M.F., Oliveira P.L., Graca-Souza A.V., Bozza M.T. (2007). Characterization of heme as activator of toll-like receptor 4. J. Biol. Chem..

[147] Nath K.A., Belcher J.D., Nath M.C., Grande J.P., Croatt A.J., Ackerman A.W., Katusic Z.S., Vercellotti G.M. (2018). Role of TLR4 signaling in the nephrotoxicity of heme and heme proteins. Am. J. Physiol. Ren. Physiol..

[148] Pradhan P., Vijayan V., Liu B., Martinez-Delgado B., Matamala N., Nikolin C., Greite R., DeLuca D.S., Janciauskiene S., Motterlini R., Foresti R., Immenschuh S. (2024). Distinct metabolic responses to heme in inflammatory human and mouse macrophages - role of nitric oxide. Redox Biol..

[149] Vallelian F., Schaer C.A., Deuel J.W., Ingoglia G., Humar R., Buehler P.W., Schaer D.J. (2018). Revisiting the putative role of heme as a trigger of inflammation. Pharmacol Res Perspect.

[150] Janciauskiene S., Vijayan V., Immenschuh S. (2020). TLR4 signaling by heme and the role of heme-binding blood proteins. Front. Immunol..

[151] Lin T., Kwak Y.H., Sammy F., He P., Thundivalappil S., Sun G., Chao W., Warren H.S. (2010). Synergistic inflammation is induced by blood degradation products with microbial toll-like receptor agonists and is blocked by hemopexin. J. Infect. Dis..

[152] Dutra F.F., Alves L.S., Rodrigues D., Fernandez P.L., de Oliveira R.B., Golenbock D.T., Zamboni D.S., Bozza M.T. (2014). Hemolysis-induced lethality involves inflammasome activation by heme. Proc. Natl. Acad. Sci. U. S. A..

[153] Frimat M., Boudhabhay I., Roumenina L.T. (2019). Hemolysis derived products toxicity and endothelium: model of the second hit. Toxins.

[154] Yamamoto M., Kensler T.W., Motohashi H. (2018). The KEAP1-NRF2 system: a thiol-based sensor-effector apparatus for maintaining redox homeostasis. Physiol. Rev..

[155] Virag L., Jaen R.I., Regdon Z., Bosca L., Prieto P. (2019). Self-defense of macrophages against oxidative injury: fighting for their own survival. Redox Biol..

[156] Wang P., Geng J., Gao J., Zhao H., Li J., Shi Y., Yang B., Xiao C., Linghu Y., Sun X., Chen X., Hong L., Qin F., Li X., Yu J.S., You H., Yuan Z., Zhou D., Johnson R.L., Chen L. (2019). Macrophage achieves self-protection against oxidative stress-induced ageing through the Mst-Nrf2 axis. Nat. Commun..

[157] McMahon M., Itoh K., Yamamoto M., Hayes J.D. (2003). Keap1-dependent proteasomal degradation of transcription factor Nrf2 contributes to the negative regulation of antioxidant response element-driven gene expression. J. Biol. Chem..

[158] Itoh K., Chiba T., Takahashi S., Ishii T., Igarashi K., Katoh Y., Oyake T., Hayashi N., Satoh K., Hatayama I., Yamamoto M., Nabeshima Y. (1997). An Nrf2/small Maf heterodimer mediates the induction of phase II detoxifying enzyme genes through antioxidant response elements. Biochem. Biophys. Res. Commun..

[159] Geng J., Sun X., Wang P., Zhang S., Wang X., Wu H., Hong L., Xie C., Li X., Zhao H., Liu Q., Jiang M., Chen Q., Zhang J., Li Y., Song S., Wang H.R., Zhou R., Johnson R.L., Chien K.Y., Lin S.C., Han J., Avruch J., Chen L., Zhou D. (2015). Kinases Mst1 and Mst2 positively regulate phagocytic induction of reactive oxygen species and bactericidal activity. Nat. Immunol..

[160] Chakraborty S., Sircar E., Bhattacharyya C., Choudhuri A., Mishra A., Dutta S., Bhatta S., Sachin K., Sengupta R. (2022). S-Denitrosylation: a crosstalk between glutathione and redoxin systems. Antioxidants.

[161] Benhar M., Forrester M.T., Stamler J.S. (2009). Protein denitrosylation: enzymatic mechanisms and cellular functions. Nat. Rev. Mol. Cell Biol..

[162] Kelleher Z.T., Sha Y., Foster M.W., Foster W.M., Forrester M.T., Marshall H.E. (2014). Thioredoxin-mediated denitrosylation regulates cytokine-induced nuclear factor kappaB (NF-kappaB) activation. J. Biol. Chem..

[163] Abbas K., Breton J., Planson A.G., Bouton C., Bignon J., Seguin C., Riquier S., Toledano M.B., Drapier J.C. (2011). Nitric oxide activates an Nrf2/sulfiredoxin antioxidant pathway in macrophages. Free Radic. Biol. Med..

[164] Nairz M., Schleicher U., Schroll A., Sonnweber T., Theurl I., Ludwiczek S., Talasz H., Brandacher G., Moser P.L., Muckenthaler M.U., Fang F.C., Bogdan C., Weiss G. (2013). Nitric oxide-mediated regulation of ferroportin-1 controls macrophage iron homeostasis and immune function in salmonella infection. J. Exp. Med..

[165] Cohen L.A., Gutierrez L., Weiss A., Leichtmann-Bardoogo Y., Zhang D.L., Crooks D.R., Sougrat R., Morgenstern A., Galy B., Hentze M.W., Lazaro F.J., Rouault T.A., Meyron-Holtz E.G. (2010). Serum ferritin is derived primarily from macrophages through a nonclassical secretory pathway. Blood.

[166] Ganz T. (2012). Macrophages and systemic iron homeostasis. J. Innate Immun..

[167] Zenke-Kawasaki Y., Dohi Y., Katoh Y., Ikura T., Ikura M., Asahara T., Tokunaga F., Iwai K., Igarashi K. (2007). Heme induces ubiquitination and degradation of the transcription factor Bach1. Mol. Cell Biol..

[168] Oyake T., Itoh K., Motohashi H., Hayashi N., Hoshino H., Nishizawa M., Yamamoto M., Igarashi K. (1996). Bach proteins belong to a novel family of BTB-basic leucine zipper transcription factors that interact with MafK and regulate transcription through the NF-E2 site. Mol. Cell Biol..

[169] Amaral E.P., Namasivayam S., Queiroz A.T.L., Fukutani E., Hilligan K.L., Aberman K., Fisher L., Bomfim C.C.B., Kauffman K., Buchanan J., Santuo L., Gazzinelli-Guimaraes P.H., Costa D.L., Teixeira M.A., Barreto-Duarte B., Rocha C.G., Santana M.F., Cordeiro-Santos M., Barber D.L., Wilkinson R.J., Kramnik I., Igarashi K., Scriba T., Mayer-Barber K.D., Andrade B.B., Sher A. (2024). BACH1 promotes tissue necrosis and Mycobacterium tuberculosis susceptibility. Nat. Microbiol..

[170] Sun J., Liu D., Jin S., Li X., Liu G., Li S., Chen F., Qin X., Zhang Y., Jiang F., Chen D., Pang Q., Hu C., Wu Y., Wang Z. (2025). Deletion of BTB and CNC homology 1 protects against staphylococcus aureus-Induced acute lung injury. J. Infect. Dis..

[171] Huang J., Zhang Y., Jiang F., Zhang Y., Li S., He S., Sun J., Chen D., Pang Q., Wu Y. (2025). Bach1 deficiency ameliorates radiation pneumonitis via activating TFAM signaling pathway. Antioxidants Redox Signal..

[172] Pradhan P., Vijayan V., Cirksena K., Buettner F.F.R., Igarashi K., Motterlini R., Foresti R., Immenschuh S. (2022). Genetic BACH1 deficiency alters mitochondrial function and increases NLRP3 inflammasome activation in mouse macrophages. Redox Biol..

[173] Carreau A., El Hafny-Rahbi B., Matejuk A., Grillon C., Kieda C. (2011). Why is the partial oxygen pressure of human tissues a crucial parameter? Small molecules and hypoxia. J. Cell Mol. Med..

[174] Atkuri K.R., Herzenberg L.A., Niemi A.K., Cowan T., Herzenberg L.A. (2007). Importance of culturing primary lymphocytes at physiological oxygen levels. Proc. Natl. Acad. Sci. U. S. A..

[175] Haas B., Chrusciel S., Fayad-Kobeissi S., Dubois-Rande J.L., Azuaje F., Boczkowski J., Motterlini R., Foresti R. (2015). Permanent culture of macrophages at physiological oxygen attenuates the antioxidant and immunomodulatory properties of dimethyl fumarate. J. Cell. Physiol..

[176] Atkuri K.R., Herzenberg L.A., Herzenberg L.A. (2005). Culturing at atmospheric oxygen levels impacts lymphocyte function. Proc. Natl. Acad. Sci. U. S. A..

[177] Stuart J.A., Fonseca J., Moradi F., Cunningham C., Seliman B., Worsfold C.R., Dolan S., Abando J., Maddalena L.A. (2018). How supraphysiological oxygen levels in standard cell culture affect oxygen-consuming reactions. Oxid. Med. Cell. Longev..

[178] Erusalimsky J.D., Moncada S. (2007). Nitric oxide and mitochondrial signaling: from physiology to pathophysiology. Arterioscler. Thromb. Vasc. Biol..

[179] Giuffre A., Sarti P., D'Itri E., Buse G., Soulimane T., Brunori M. (1996). On the mechanism of inhibition of cytochrome c oxidase by nitric oxide. J. Biol. Chem..

[180] Brunori M., Giuffre A., Forte E., Mastronicola D., Barone M.C., Sarti P. (2004). Control of cytochrome c oxidase activity by nitric oxide. Biochim. Biophys. Acta.

[181] Mason M.G., Nicholls P., Wilson M.T., Cooper C.E. (2006). Nitric oxide inhibition of respiration involves both competitive (heme) and noncompetitive (copper) binding to cytochrome c oxidase. Proc. Natl. Acad. Sci. U. S. A..

[182] Brown G.C., Cooper C.E. (1994). Nanomolar concentrations of nitric oxide reversibly inhibit synaptosomal respiration by competing with oxygen at cytochrome oxidase. FEBS Lett..

[183] Cleeter M.W., Cooper J.M., Darley-Usmar V.M., Moncada S., Schapira A.H. (1994). Reversible inhibition of cytochrome c oxidase, the terminal enzyme of the mitochondrial respiratory chain, by nitric oxide. Implications for neurodegenerative diseases. FEBS Lett..

[184] Schweizer M., Richter C. (1994). Nitric oxide potently and reversibly deenergizes mitochondria at low oxygen tension. Biochem. Biophys. Res. Commun..

[185] Viola A., Munari F., Sanchez-Rodriguez R., Scolaro T., Castegna A. (2019). The metabolic signature of macrophage responses. Front. Immunol..

[186] Cooper C.E., Giulivi C. (2007). Nitric oxide regulation of mitochondrial oxygen consumption II: molecular mechanism and tissue physiology. Am. J. Physiol. Cell Physiol..

[187] Baseler W.A., Davies L.C., Quigley L., Ridnour L.A., Weiss J.M., Hussain S.P., Wink D.A., McVicar D.W. (2016). Autocrine IL-10 functions as a rheostat for M1 macrophage glycolytic commitment by tuning nitric oxide production. Redox Biol..

[188] Van den Bossche J., Baardman J., Otto N.A., van der Velden S., Neele A.E., van den Berg S.M., Luque-Martin R., Chen H.J., Boshuizen M.C., Ahmed M., Hoeksema M.A., de Vos A.F., de Winther M.P. (2016). Mitochondrial dysfunction prevents repolarization of inflammatory macrophages. Cell Rep..

[189] Mao K., Chen S., Chen M., Ma Y., Wang Y., Huang B., He Z., Zeng Y., Hu Y., Sun S., Li J., Wu X., Wang X., Strober W., Chen C., Meng G., Sun B. (2013). Nitric oxide suppresses NLRP3 inflammasome activation and protects against LPS-induced septic shock. Cell Res..

[190] Bailey J.D., Diotallevi M., Nicol T., McNeill E., Shaw A., Chuaiphichai S., Hale A., Starr A., Nandi M., Stylianou E., McShane H., Davis S., Fischer R., Kessler B.M., McCullagh J., Channon K.M., Crabtree M.J. (2019). Nitric oxide modulates metabolic remodeling in inflammatory macrophages through TCA cycle regulation and itaconate accumulation. Cell Rep..

[191] Palmieri E.M., Gonzalez-Cotto M., Baseler W.A., Davies L.C., Ghesquiere B., Maio N., Rice C.M., Rouault T.A., Cassel T., Higashi R.M., Lane A.N., Fan T.W., Wink D.A., McVicar D.W. (2020). Nitric oxide orchestrates metabolic rewiring in M1 macrophages by targeting aconitase 2 and pyruvate dehydrogenase. Nat. Commun..

[192] Brown G.C. (1999). Nitric oxide and mitochondrial respiration. Biochim. Biophys. Acta.

[193] Radi R., Rodriguez M., Castro L., Telleri R. (1994). Inhibition of mitochondrial electron transport by peroxynitrite. Arch. Biochem. Biophys..

[194] Seim G.L., John S.V., Arp N.L., Fang Z., Pagliarini D.J., Fan J. (2023). Nitric oxide-driven modifications of lipoic arm inhibit alpha-ketoacid dehydrogenases. Nat. Chem. Biol..

[195] Abu Shelbayeh O., Arroum T., Morris S., Busch K.B. (2023). PGC-1alpha is a master regulator of mitochondrial lifecycle and ROS stress response. Antioxidants.

[196] Lira V.A., Brown D.L., Lira A.K., Kavazis A.N., Soltow Q.A., Zeanah E.H., Criswell D.S. (2010). Nitric oxide and AMPK cooperatively regulate PGC-1 in skeletal muscle cells. J. Physiol..

[197] Nisoli E., Falcone S., Tonello C., Cozzi V., Palomba L., Fiorani M., Pisconti A., Brunelli S., Cardile A., Francolini M., Cantoni O., Carruba M.O., Moncada S., Clementi E. (2004). Mitochondrial biogenesis by NO yields functionally active mitochondria in mammals. Proc. Natl. Acad. Sci. U. S. A..

[198] Cherry A.D., Piantadosi C.A. (2015). Regulation of mitochondrial biogenesis and its intersection with inflammatory responses. Antioxidants Redox Signal..

[199] Hortelano S., Alvarez A.M., Bosca L. (1999). Nitric oxide induces tyrosine nitration and release of cytochrome c preceding an increase of mitochondrial transmembrane potential in macrophages. FASEB J..

[200] Gall T., Petho D., Erdelyi K., Egri V., Balla J.G., Nagy A., Nagy A., Poliska S., Gram M., Gabriel R., Nagy P., Balla J., Balla G. (2024). Heme: a link between hemorrhage and retinopathy of prematurity progression. Redox Biol..

[201] Sharma R., Antypiuk A., Vance S.Z., Manwani D., Pearce Q., Cox J.E., An X., Yazdanbakhsh K., Vinchi F. (2023). Macrophage metabolic rewiring improves heme-suppressed efferocytosis and tissue damage in sickle cell disease. Blood.

[202] Li T., Adams J., Zhu P., Zhang T., Tu F., Gravitte A., Zhang X., Liu L., Casteel J., Yakubenko V., Williams D.L., Li C., Wang X. (2025). The role of heme in sepsis induced Kupffer cell PANoptosis and senescence. Cell Death Dis..

[203] Prestes E.B., Alves L.S., Rodrigues D.A.S., Dutra F.F., Fernandez P.L., Paiva C.N., Kagan J.C., Bozza M.T. (2020). Mitochondrial reactive oxygen species participate in signaling triggered by heme in macrophages and upon hemolysis. J. Immunol..

[204] Rock K.L., Kono H. (2008). The inflammatory response to cell death. Annu. Rev. Pathol..

[205] Galluzzi L., Vitale I., Aaronson S.A., Abrams J.M., Adam D., Agostinis P., Alnemri E.S., Altucci L., Amelio I., Andrews D.W., Annicchiarico-Petruzzelli M., Antonov A.V., Arama E., Baehrecke E.H., Barlev N.A., Bazan N.G., Bernassola F., Bertrand M.J.M., Bianchi K., Blagosklonny M.V., Blomgren K., Borner C., Boya P., Brenner C., Campanella M., Candi E., Carmona-Gutierrez D., Cecconi F., Chan F.K., Chandel N.S., Cheng E.H., Chipuk J.E., Cidlowski J.A., Ciechanover A., Cohen G.M., Conrad M., Cubillos-Ruiz J.R., Czabotar P.E., D'Angiolella V., Dawson T.M., Dawson V.L., De Laurenzi V., De Maria R., Debatin K.M., DeBerardinis R.J., Deshmukh M., Di Daniele N., Di Virgilio F., Dixit V.M., Dixon S.J., Duckett C.S., Dynlacht B.D., El-Deiry W.S., Elrod J.W., Fimia G.M., Fulda S., Garcia-Saez A.J., Garg A.D., Garrido C., Gavathiotis E., Golstein P., Gottlieb E., Green D.R., Greene L.A., Gronemeyer H., Gross A., Hajnoczky G., Hardwick J.M., Harris I.S., Hengartner M.O., Hetz C., Ichijo H., Jaattela M., Joseph B., Jost P.J., Juin P.P., Kaiser W.J., Karin M., Kaufmann T., Kepp O., Kimchi A., Kitsis R.N., Klionsky D.J., Knight R.A., Kumar S., Lee S.W., Lemasters J.J., Levine B., Linkermann A., Lipton S.A., Lockshin R.A., Lopez-Otin C., Lowe S.W., Luedde T., Lugli E., MacFarlane M., Madeo F., Malewicz M., Malorni W., Manic G., Marine J.C., Martin S.J., Martinou J.C., Medema J.P., Mehlen P., Meier P., Melino S., Miao E.A., Molkentin J.D., Moll U.M., Munoz-Pinedo C., Nagata S., Nunez G., Oberst A., Oren M., Overholtzer M., Pagano M., Panaretakis T., Pasparakis M., Penninger J.M., Pereira D.M., Pervaiz S., Peter M.E., Piacentini M., Pinton P., Prehn J.H.M., Puthalakath H., Rabinovich G.A., Rehm M., Rizzuto R., Rodrigues C.M.P., Rubinsztein D.C., Rudel T., Ryan K.M., Sayan E., Scorrano L., Shao F., Shi Y., Silke J., Simon H.U., Sistigu A., Stockwell B.R., Strasser A., Szabadkai G., Tait S.W.G., Tang D., Tavernarakis N., Thorburn A., Tsujimoto Y., Turk B., Vanden Berghe T., Vandenabeele P., Vander Heiden M.G., Villunger A., Virgin H.W., Vousden K.H., Vucic D., Wagner E.F., Walczak H., Wallach D., Wang Y., Wells J.A., Wood W., Yuan J., Zakeri Z., Zhivotovsky B., Zitvogel L., Melino G., Kroemer G. (2018). Molecular mechanisms of cell death: recommendations of the nomenclature committee on cell death 2018. Cell Death Differ..

[206] Kroemer G., Galassi C., Zitvogel L., Galluzzi L. (2022). Immunogenic cell stress and death. Nat. Immunol..

[207] Marchi S., Guilbaud E., Tait S.W.G., Yamazaki T., Galluzzi L. (2023). Mitochondrial control of inflammation. Nat. Rev. Immunol..

[208] Kerr J.F., Wyllie A.H., Currie A.R. (1972). Apoptosis: a basic biological phenomenon with wide-ranging implications in tissue kinetics. Br. J. Cancer.

[209] Bock F.J., Riley J.S. (2023). When cell death goes wrong: inflammatory outcomes of failed apoptosis and mitotic cell death. Cell Death Differ..

[210] Aufschnaiter A., Oh T.J., Oberst A. (2026). The landscape of regulated cell death: it's all downhill from here. Mol. Cell.

[211] Kaczmarek A., Vandenabeele P., Krysko D.V. (2013). Necroptosis: the release of damage-associated molecular patterns and its physiological relevance. Immunity.

[212] Roh J.S., Sohn D.H. (2018). Damage-associated molecular patterns in inflammatory diseases. Immune Netw..

[213] Sundaram B., Pandian N., Mall R., Wang Y., Sarkar R., Kim H.J., Malireddi R.K.S., Karki R., Janke L.J., Vogel P., Kanneganti T.D. (2023). NLRP12-PANoptosome activates PANoptosis and pathology in response to heme and PAMPs. Cell.

[214] Chae I.H., Park K.W., Kim H.S., Oh B.H. (2004). Nitric oxide-induced apoptosis is mediated by Bax/Bcl-2 gene expression, transition of cytochrome c, and activation of caspase-3 in rat vascular smooth muscle cells. Clin. Chim. Acta.

[215] Snyder C.M., Shroff E.H., Liu J., Chandel N.S. (2009). Nitric oxide induces cell death by regulating anti-apoptotic BCL-2 family members. PLoS One.

[216] Brune B., Schneiderhan N. (2003). Nitric oxide evoked p53-accumulation and apoptosis. Toxicol. Lett..

[217] Marshall H.E., Stamler J.S. (2002). Nitrosative stress-induced apoptosis through inhibition of NF-kappa B. J. Biol. Chem..

[218] Li D.Y., Tao L., Liu H., Christopher T.A., Lopez B.L., Ma X.L. (2006). Role of ERK1/2 in the anti-apoptotic and cardioprotective effects of nitric oxide after myocardial ischemia and reperfusion. Apoptosis.

[219] Matsuzaki H., Tamatani M., Mitsuda N., Namikawa K., Kiyama H., Miyake S., Tohyama M. (1999). Activation of Akt kinase inhibits apoptosis and changes in Bcl-2 and Bax expression induced by nitric oxide in primary hippocampal neurons. J. Neurochem..

[220] Homma T., Kobayashi S., Conrad M., Konno H., Yokoyama C., Fujii J. (2021). Nitric oxide protects against ferroptosis by aborting the lipid peroxidation chain reaction. Nitric Oxide.

[221] Mikulska-Ruminska K., Anthonymuthu T.S., Levkina A., Shrivastava I.H., Kapralov A.A., Bayir H., Kagan V.E., Bahar I. (2021). NO(●) represses the oxygenation of arachidonoyl PE by 15LOX/PEBP1: mechanism and role in ferroptosis. Int. J. Mol. Sci..

[222] Rubbo H., Radi R., Anselmi D., Kirk M., Barnes S., Butler J., Eiserich J.P., Freeman B.A. (2000). Nitric oxide reaction with lipid peroxyl radicals spares alpha-tocopherol during lipid peroxidation. Greater oxidant protection from the pair nitric oxide/alpha-tocopherol than alpha-tocopherol/ascorbate. J. Biol. Chem..

[223] Fortes G.B., Alves L.S., de Oliveira R., Dutra F.F., Rodrigues D., Fernandez P.L., Souto-Padron T., De Rosa M.J., Kelliher M., Golenbock D., Chan F.K., Bozza M.T. (2012). Heme induces programmed necrosis on macrophages through autocrine TNF and ROS production. Blood.

[224] Kwon M.Y., Park E., Lee S.J., Chung S.W. (2015). Heme oxygenase-1 accelerates erastin-induced ferroptotic cell death. Oncotarget.

[225] Chang L.C., Chiang S.K., Chen S.E., Yu Y.L., Chou R.H., Chang W.C. (2018). Heme oxygenase-1 mediates BAY 11-7085 induced ferroptosis. Cancer Lett..

[226] Li R., Wei R., Liu C., Zhang K., He S., Liu Z., Huang J., Tang Y., An Q., Lin L., Gan L., Zhao L., Zou X., Wang F., Ping Y., Ma Q. (2024). Heme oxygenase 1-mediated ferroptosis in Kupffer cells initiates liver injury during heat stroke. Acta Pharm. Sin. B.

[227] Malireddi R.K.S., Kesavardhana S., Kanneganti T.D. (2019). ZBP1 and TAK1: master regulators of NLRP3 inflammasome/pyroptosis, apoptosis, and necroptosis (PAN-optosis). Front. Cell. Infect. Microbiol..

[228] Albina J.E., Cui S., Mateo R.B., Reichner J.S. (1993). Nitric oxide-mediated apoptosis in murine peritoneal macrophages. J. Immunol..

[229] Sarih M., Souvannavong V., Adam A. (1993). Nitric oxide synthase induces macrophage death by apoptosis. Biochem. Biophys. Res. Commun..

[230] Brune B. (2003). Nitric oxide: NO apoptosis or turning it ON?. Cell Death Differ..

[231] Kim Y.M., Bombeck C.A., Billiar T.R. (1999). Nitric oxide as a bifunctional regulator of apoptosis. Circ. Res..

[232] Li J., Bombeck C.A., Yang S., Kim Y.M., Billiar T.R. (1999). Nitric oxide suppresses apoptosis via interrupting caspase activation and mitochondrial dysfunction in cultured hepatocytes. J. Biol. Chem..

[233] Rossig L., Fichtlscherer B., Breitschopf K., Haendeler J., Zeiher A.M., Mulsch A., Dimmeler S. (1999). Nitric oxide inhibits caspase-3 by S-nitrosation in vivo. J. Biol. Chem..

[234] Jacotot E., Costantini P., Laboureau E., Zamzami N., Susin S.A., Kroemer G. (1999). Mitochondrial membrane permeabilization during the apoptotic process. Ann. N. Y. Acad. Sci..

[235] Hortelano S., Dallaporta B., Zamzami N., Hirsch T., Susin S.A., Marzo I., Bosca L., Kroemer G. (1997). Nitric oxide induces apoptosis via triggering mitochondrial permeability transition. FEBS Lett..

[236] Gotoh T., Oyadomari S., Mori K., Mori M. (2002). Nitric oxide-induced apoptosis in RAW 264.7 macrophages is mediated by endoplasmic reticulum stress pathway involving ATF6 and CHOP. J. Biol. Chem..

[237] Gall T., Petho D., Nagy A., Hendrik Z., Mehes G., Potor L., Gram M., Akerstrom B., Smith A., Nagy P., Balla G., Balla J. (2018). Heme induces endoplasmic reticulum stress (HIER stress) in human aortic smooth muscle cells. Front. Physiol..

[238] Petho D., Hendrik Z., Nagy A., Beke L., Patsalos A., Nagy L., Poliska S., Mehes G., Toth C., Potor L., Eaton J.W., Jacob H.S., Balla G., Balla J., Gall T. (2021). Heme cytotoxicity is the consequence of endoplasmic reticulum stress in atherosclerotic plaque progression. Sci. Rep..

[239] Gall T., Balla G., Balla J. (2019). Heme, heme oxygenase, and endoplasmic reticulum Stress-A new insight into the pathophysiology of vascular diseases. Int. J. Mol. Sci..

[240] Nishizawa H., Yamanaka M., Igarashi K. (2023). Ferroptosis: regulation by competition between NRF2 and BACH1 and propagation of the death signal. FEBS J..

[241] Kapralov A.A., Yang Q., Dar H.H., Tyurina Y.Y., Anthonymuthu T.S., Kim R., St Croix C.M., Mikulska-Ruminska K., Liu B., Shrivastava I.H., Tyurin V.A., Ting H.C., Wu Y.L., Gao Y., Shurin G.V., Artyukhova M.A., Ponomareva L.A., Timashev P.S., Domingues R.M., Stoyanovsky D.A., Greenberger J.S., Mallampalli R.K., Bahar I., Gabrilovich D.I., Bayir H., Kagan V.E. (2020). Redox lipid reprogramming commands susceptibility of macrophages and microglia to ferroptotic death. Nat. Chem. Biol..

[242] Dar H.H., Anthonymuthu T.S., Ponomareva L.A., Souryavong A.B., Shurin G.V., Kapralov A.O., Tyurin V.A., Lee J.S., Mallampalli R.K., Wenzel S.E., Bayir H., Kagan V.E. (2021). A new thiol-independent mechanism of epithelial host defense against Pseudomonas aeruginosa: inos/no(∗) sabotage of theft-ferroptosis. Redox Biol..

[243] Takabayashi A., Kawai Y., Iwata S., Kanai M., Denno R., Kawada K., Obama K., Taki Y. (2000). Nitric oxide induces a decrease in the mitochondrial membrane potential of peripheral blood lymphocytes, especially in natural killer cells. Antioxidants Redox Signal..

[244] Mestas J., Hughes C.C. (2004). Of mice and not men: differences between mouse and human immunology. J. Immunol..

[245] Seok J., Warren H.S., Cuenca A.G., Mindrinos M.N., Baker H.V., Xu W., Richards D.R., McDonald-Smith G.P., Gao H., Hennessy L., Finnerty C.C., Lopez C.M., Honari S., Moore E.E., Minei J.P., Cuschieri J., Bankey P.E., Johnson J.L., Sperry J., Nathens A.B., Billiar T.R., West M.A., Jeschke M.G., Klein M.B., Gamelli R.L., Gibran N.S., Brownstein B.H., Miller-Graziano C., Calvano S.E., Mason P.H., Cobb J.P., Rahme L.G., Lowry S.F., Maier R.V., Moldawer L.L., Herndon D.N., Davis R.W., Xiao W., Tompkins R.G. (2013). Genomic responses in mouse models poorly mimic human inflammatory diseases. Proc. Natl. Acad. Sci. USA.

[246] Schroder K., Irvine K.M., Taylor M.S., Bokil N.J., Le Cao K.A., Masterman K.A., Labzin L.I., Semple C.A., Kapetanovic R., Fairbairn L., Akalin A., Faulkner G.J., Baillie J.K., Gongora M., Daub C.O., Kawaji H., McLachlan G.J., Goldman N., Grimmond S.M., Carninci P., Suzuki H., Hayashizaki Y., Lenhard B., Hume D.A., Sweet M.J. (2012). Conservation and divergence in toll-like receptor 4-regulated gene expression in primary human versus mouse macrophages. Proc. Natl. Acad. Sci. U. S. A.

[247] Vijayan V., Pradhan P., Braud L., Fuchs H.R., Gueler F., Motterlini R., Foresti R., Immenschuh S. (2019). Human and murine macrophages exhibit differential metabolic responses to lipopolysaccharide - a divergent role for glycolysis. Redox Biol..

[248] Schneemann M., Schoedon G., Hofer S., Blau N., Guerrero L., Schaffner A. (1993). Nitric oxide synthase is not a constituent of the antimicrobial armature of human mononuclear phagocytes. J. Infect. Dis..

[249] Weinberg J.B., Misukonis M.A., Shami P.J., Mason S.N., Sauls D.L., Dittman W.A., Wood E.R., Smith G.K., McDonald B., Bachus K.E. (1995). Human mononuclear phagocyte inducible nitric oxide synthase (iNOS): analysis of iNOS mRNA, iNOS protein, biopterin, and nitric oxide production by blood monocytes and peritoneal macrophages. Blood.

[250] Murray P.J., Wynn T.A. (2011). Obstacles and opportunities for understanding macrophage polarization. J. Leukoc. Biol..

[251] Palmieri E.M., McGinity C., Wink D.A., McVicar D.W. (2020). Nitric oxide in macrophage immunometabolism: hiding in plain sight. Metabolites.

[252] Heuser S.K., Li J., Li Z., LoBue A., Heard K., Hocks J., Suvorava T., Cadeddu R.P., Strupp C., Dunaway L., Zhuge Z., Gelhaus S.L., Heinen A., Germing U., Feelisch M., Carlstrom M., Isakson B., Kelm M., Lundberg J.O., Cortese-Krott M.M. (2025). Divergent roles of red cell arginase in humans and mice: RBC Arg1 KO mice show preserved systemic l-arginine bioavailability and infarct size in vivo. Redox Biol..

[253] Drehmer D., Mesquita Luiz J.P., Hernandez C.A.S., Alves-Filho J.C., Hussell T., Townsend P.A., Moncada S. (2022). Nitric oxide favours tumour-promoting inflammation through mitochondria-dependent and -independent actions on macrophages. Redox Biol..

[254] Spiller K.L., Wrona E.A., Romero-Torres S., Pallotta I., Graney P.L., Witherel C.E., Panicker L.M., Feldman R.A., Urbanska A.M., Santambrogio L., Vunjak-Novakovic G., Freytes D.O. (2016). Differential gene expression in human, murine, and cell line-derived macrophages upon polarization. Exp. Cell Res..

[255] Sun J., Li N., Oh K.S., Dutta B., Vayttaden S.J., Lin B., Ebert T.S., De Nardo D., Davis J., Bagirzadeh R., Lounsbury N.W., Pasare C., Latz E., Hornung V., Fraser I.D. (2016). Comprehensive RNAi-based screening of human and mouse TLR pathways identifies species-specific preferences in signaling protein use. Sci. Signal..

